# Supplementary material for: Regulation of AP1 adaptor assembly by the bi-handed chaperone MEA1
Source: Nat Commun. 2026 Jan 20;17:1876. doi: 10.1038/s41467-026-68662-3 (PMC12923701; doi:10.1038/s41467-026-68662-3)
Supplement: Supplementary file 1 — Supplementary Information [file 41467_2026_68662_MOESM1_ESM.pdf]

Supplementary Information

Regulation of AP1 adaptor assembly by the bi-handed chaperone MEA1

Chun Wan[1], Jingyi Wu[1], Yan Ouyang[1], Harrison Puscher[1†], Yuan Tian[2], Suzhao Li[3], Qian Yin[2*], and Jingshi Shen[1*]

[1]Department of Molecular, Cellular and Developmental Biology, University of Colorado, Boulder, CO 80309, USA.
[2]Department of Biological Sciences and Institute of Molecular Biophysics, Florida State University, Tallahassee, FL 32306, USA.
[3]Department of Medicine, University of Colorado Anschutz Medical Campus, Aurora, CO 80045, USA.
[†]Present address: Department of Biological Sciences, University of Southern California, Los Angeles, CA 90089, USA.
[*]Correspondence: yin@bio.fsu.edu (Q.Y.); jingshi.shen@colorado.edu (J.S.)

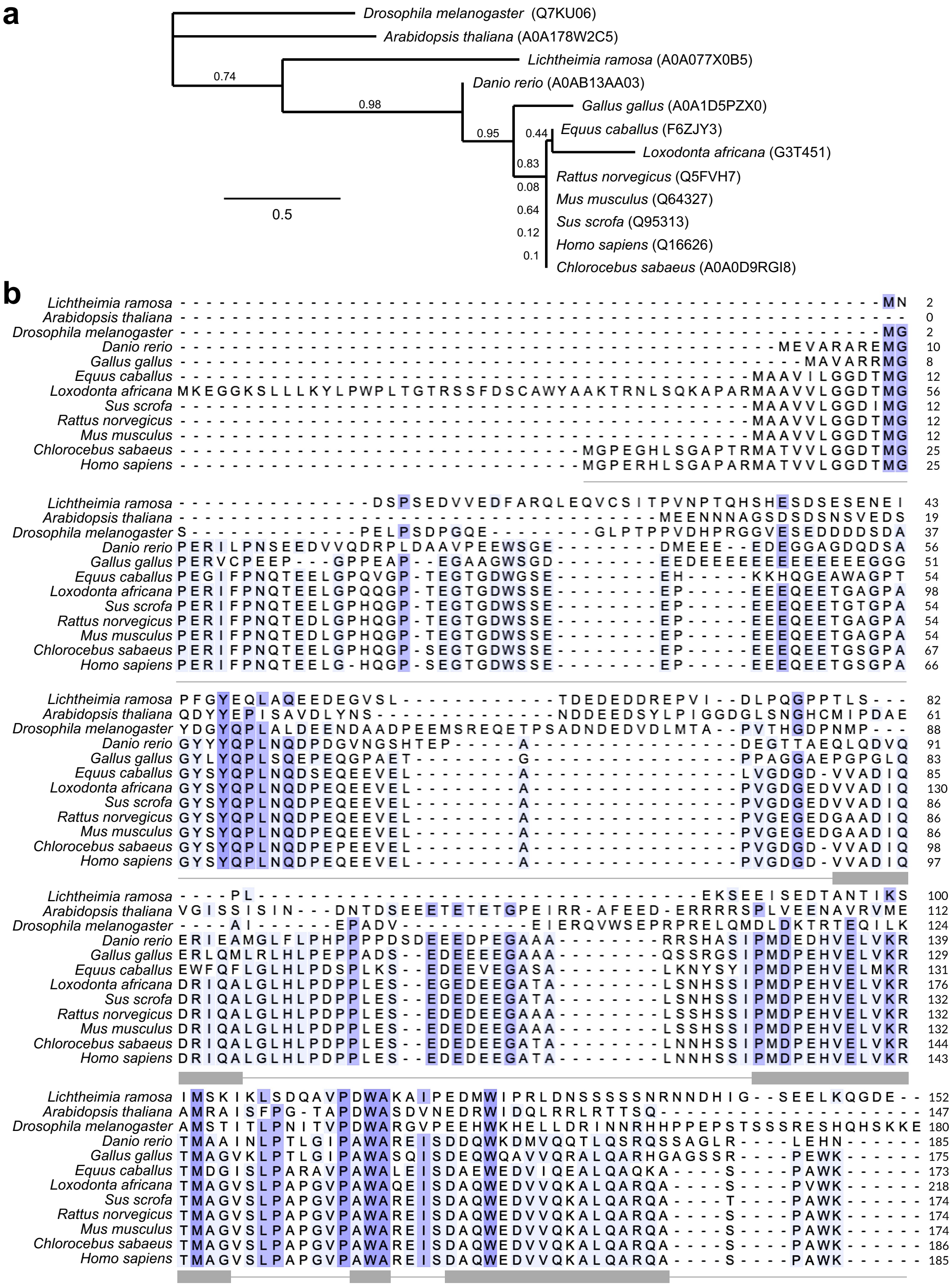

Figure S1. Evolutionary conservation of MEA1. (a) Phylogenetic analysis of MEA1 proteins from representative species. Sequences (with UniProt identifiers) were aligned using MUSCLE and the phylogenetic tree was reconstructed using the maximum likelihood method implemented in the PhyML program, and a bootstrapping procedure was used for branch support. Bootstrap values from PhyML are shown on branching points. The branch length is proportional to the number of substitutions per site. All steps were performed using Phylogeny.fr

(http://phylogeny.lirmm.fr). **(b)** Alignment of MEA1 protein sequences from representative species. The alignment was performed using Clustal Omega with default settings. Conserved residues are highlighted in blue with a darker color corresponding to a higher degree of conservation. The secondary structure of human MEA1, predicted by AlphaFold (AF-Q16626-F1), is annotated below the protein sequence, with cylinders representing α-helices. Related to Figure 1.

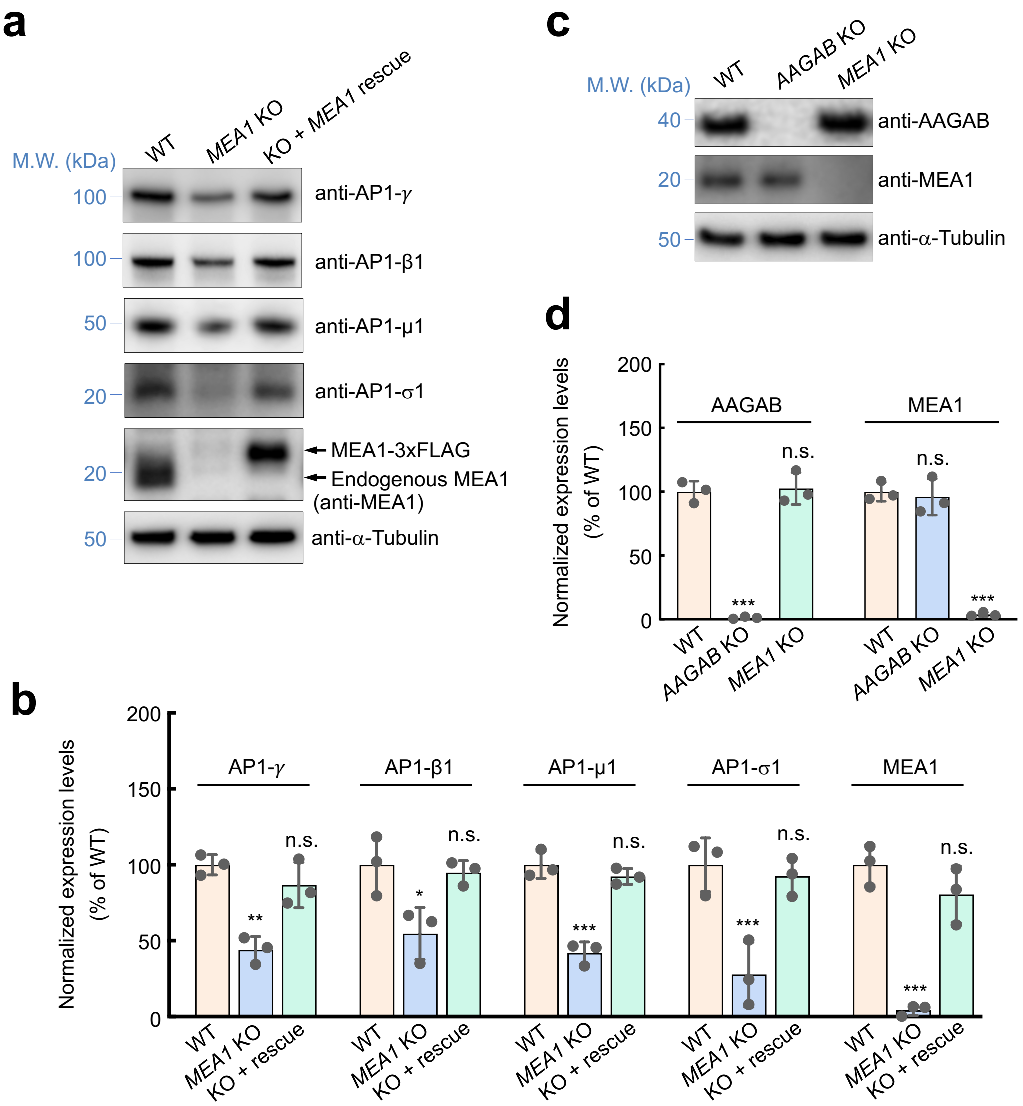

Figure S2. AP1 expression is diminished in MEA1-deficient cells. (a) Representative immunoblots showing the expression of the indicated proteins in wild-type (WT) RPE1 cells, *MEA1* KO cells, or *MEA1* KO cells expressing MEA1-3xFLAG. **(b)** Quantification of protein expression in the indicated cell lines based on immunoblots in panel a. In this figure, data normalization was performed by setting the mean value of WT data points as 100% and all data points including WT ones were normalized to that mean value. Data are presented as mean ± SD of three biological replicates. *** $P < 0.001$; ** $P < 0.01$; * $P < 0.05$; n.s., $P > 0.05$ (two-way ANOVA). Based on the strong reduction of σ1 expression in *MEA1* KO cells and the known interdependence of AP1 subunits, most residual γ, β1, and μ1 proteins in *MEA1* KO cells were likely present in nonfunctional, unassembled forms. **(c)** Representative immunoblots showing the expression of the indicated proteins in WT, *AAGAB* KO, and *MEA1* KO HeLa cells. **(d)** Quantification of protein expression in the indicated cell lines based on immunoblots in panel c. Data are presented as mean ± SD of three biological replicates. *** $P < 0.001$; n.s., $P > 0.05$ (two-way ANOVA). Related to Figure 3.

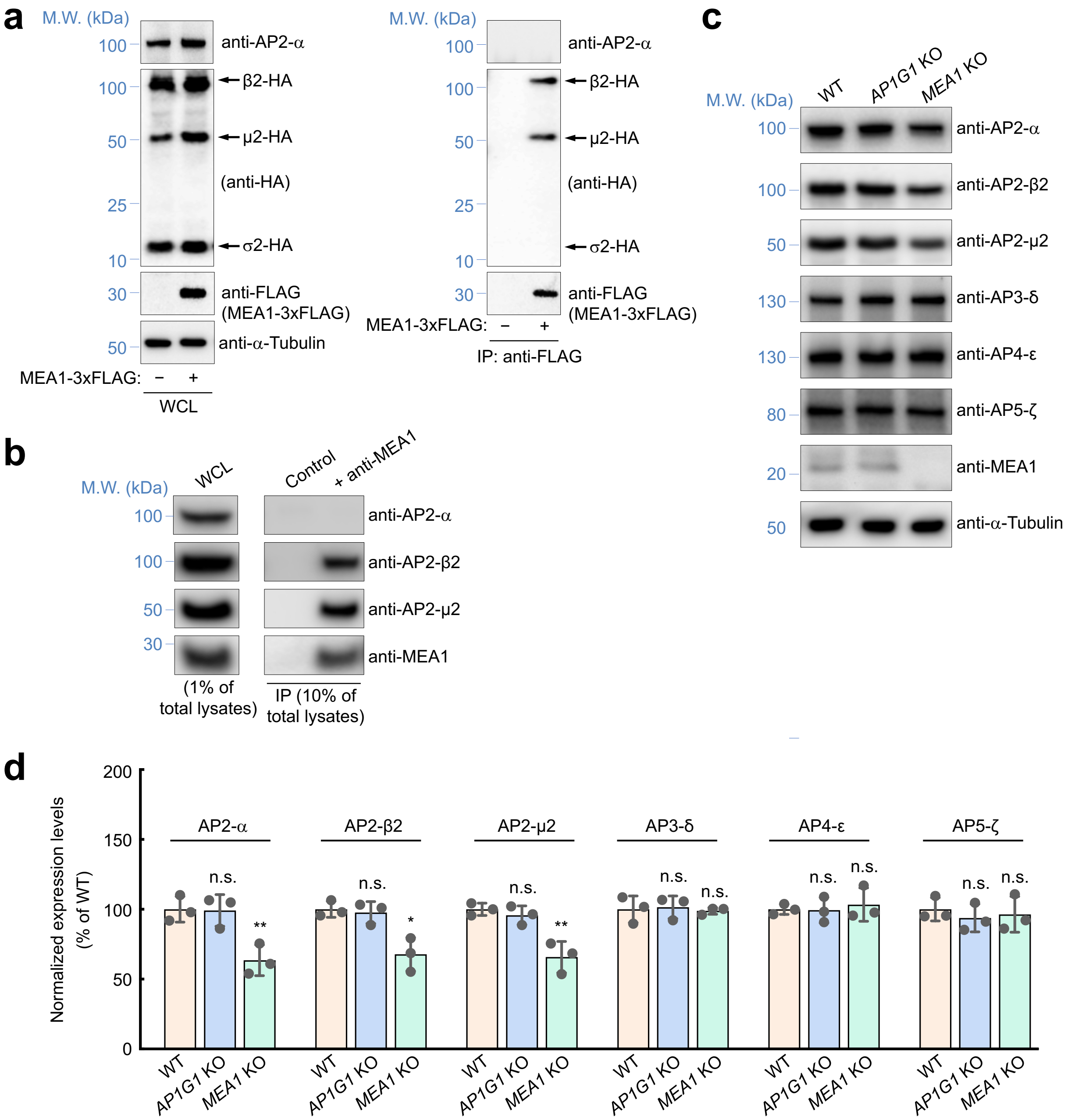

Figure S3. MEA1 primarily regulates AP1. (a) Representative immunoblots showing the interaction of 3xFLAG-tagged MEA1 with HA-tagged AP2 subunits (except α, which was untagged). Plasmids encoding AP2 subunits were transiently expressed in HEK 293T cells with either an empty vector or a plasmid encoding 3xFLAG-tagged MEA1. MEA1 was immunoprecipitated using anti-FLAG antibodies, and proteins in the immunoprecipitates were detected by immunoblotting. **(b)** Representative immunoblots showing the interactions between endogenous MEA1 and AP2 subunits. MEA1 and associated proteins were immunoprecipitated from WT HEK 293T cells using anti-MEA1 antibodies and protein A/G beads. Proteins present in the immunoprecipitates were detected by immunoblotting. Endogenous σ2 was not detected by immunoblotting. **(c)** Representative immunoblots showing the expression levels of the indicated

proteins in WT and KO HeLa cells. **(d)** Quantification of protein expression in WT and KO HeLa cells based on immunoblots in (c). Data normalization was performed by setting the mean value of WT data points as 100% and all data points including WT ones were normalized to that mean value. Data are presented as mean ± SD of three biological replicates. ** $P < 0.001$; * $P < 0.05$; n.s., $P > 0.05$ (two-way ANOVA). Related to Figure 3.

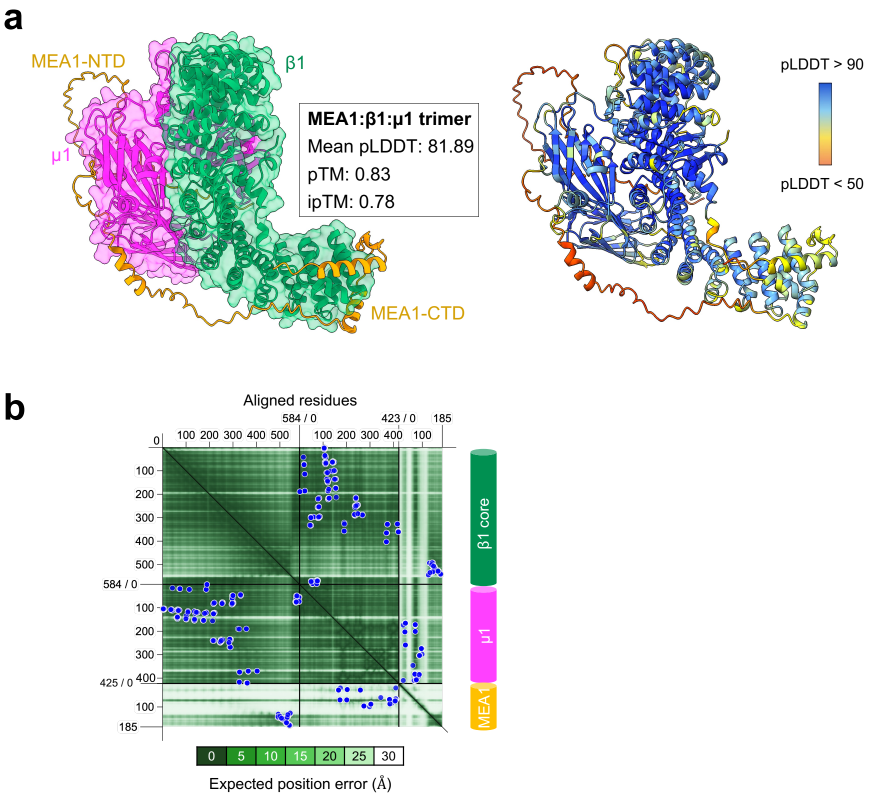

Figure S4. Structural model of MEA1:β1:µ1 trimer. (a) AlphaFold-predicted structure of the MEA1:β1:µ1 trimer, shown with coloring by protein subunits (left) and by pLDDT scores (right). The prediction was performed using full-length (FL) MEA1, the core domain of β1 (a.a. 1-584), and FL µ1 as input. The CIF file of the predicted structure is included in Supplementary Data 2. **(b)** PAE heatmap of the AlphaFold-predicted structure of the MEA1:β1:µ1 trimer shown in panel a. Interchain interactions are represented as blue dots. Related to Figure 5.

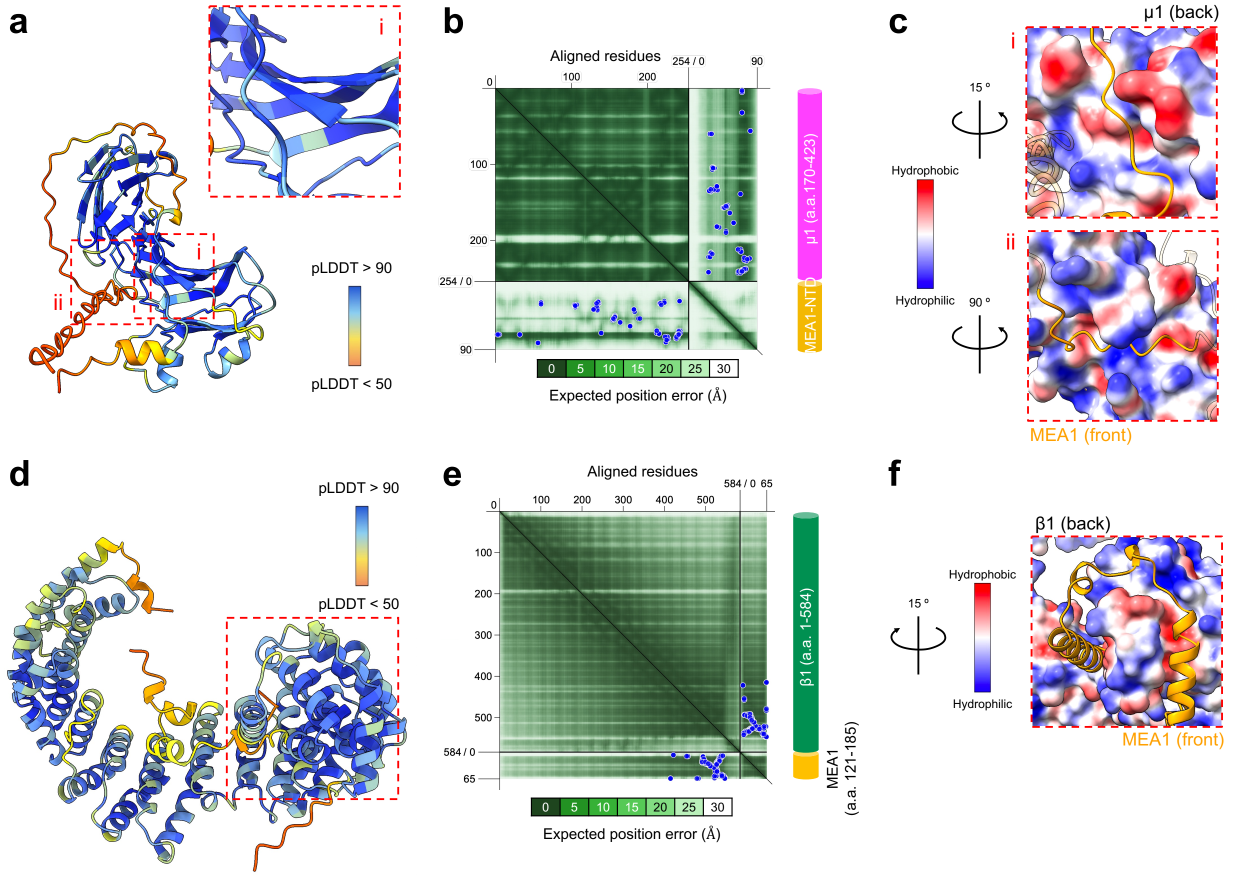

Figure S5. Structural models of MEA1-NTD:µ1 and MEA1-CTD:β1 dimers. (a) AlphaFold-predicted structure of the MEA1-NTD:µ1 dimer, colored by pLDDT confidence scores. Inset: magnified view of the MEA1-NTD:µ1 binding interface (dashed box). The structural model colored by protein subunits is shown in Figure 5a. **(b)** Predicted aligned error (PAE) heatmap of the MEA1-NTD:µ1 dimer structure shown in panel a and Figure 5a. **(c)** Structural model showing hydrophobic regions of the µ1 subunit shielded by MEA1-NTD (a.a. 1-90), corresponding to the dashed box in panel a, shown with the indicated rotation. **(d)** AlphaFold-predicted structure of the MEA1-CTD:β1 dimer, colored by pLDDT scores. The subunit-colored version of this model is shown in Figure 5c. **(e)** PAE heatmap of the MEA1-CTD:β1 dimer structure shown in panel d and Figure 5c. **(f)** Structural model showing hydrophobic regions of the β1 subunit shielded by MEA1-CTD (a.a. 121-185), corresponding to the dashed box in panel d, shown with the indicated rotation. Related to Figure 5.

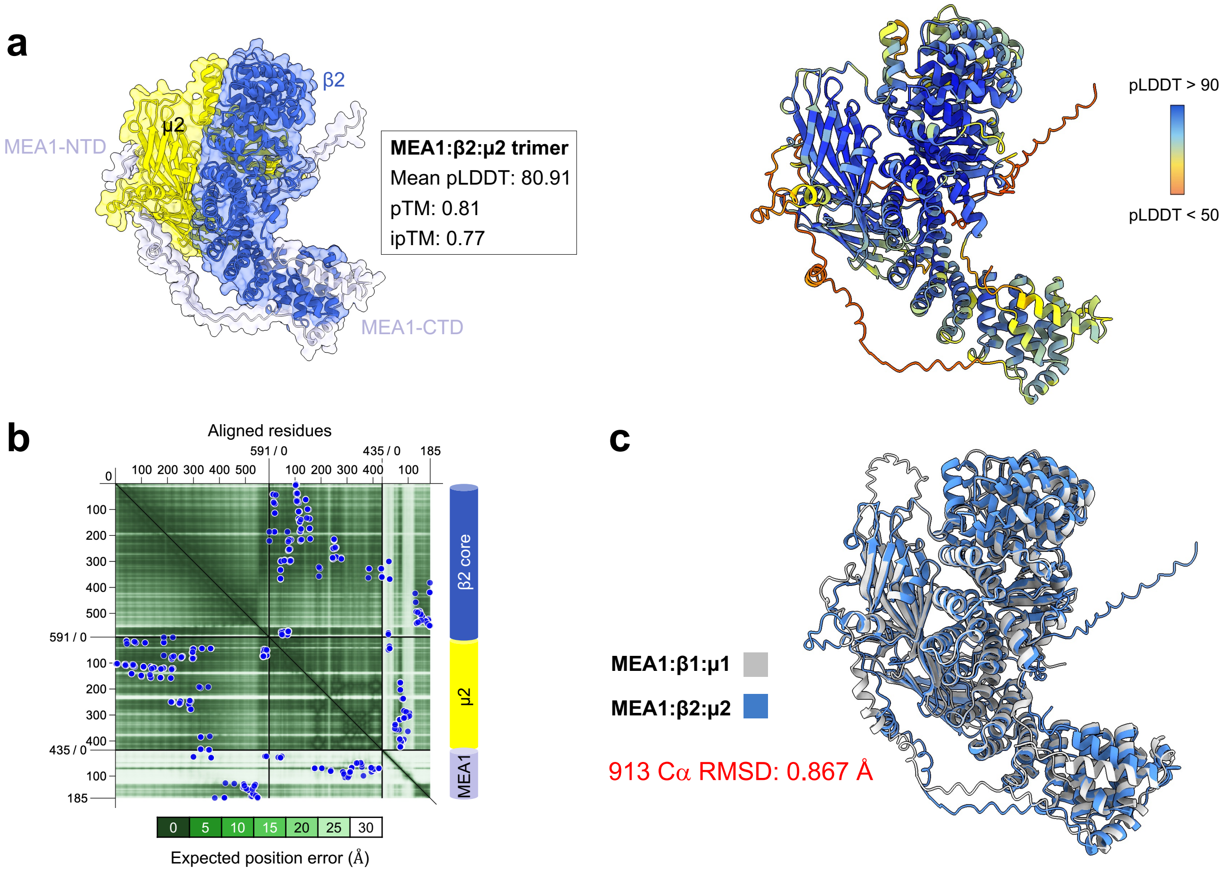

Figure S6. Structural model of the MEA1:β2:µ2 trimer. (a) AlphaFold-predicted structure of the MEA1:β2:µ2 trimer, shown colored by protein subunits (left) and by pLDDT scores (right). The prediction was generated using FL MEA1, the core domain of β2 (a.a. 1-591), and FL µ2 as input. The CIF file of the predicted model is provided in Supplementary Data 5. **(b)** PAE heatmap corresponding to the AlphaFold-predicted structure shown in panel a; interchain interactions are indicated by blue dots. **(c)** Superposition of the AlphaFold-predicted MEA1:β1:µ1 trimer (shown in Figure S4) and the MEA1:β2:µ2 trimer. The RMSD between 913 pruned atom pairs is 0.867 Å (11.988 Å across all 1188 pairs). Related to Figure 5.

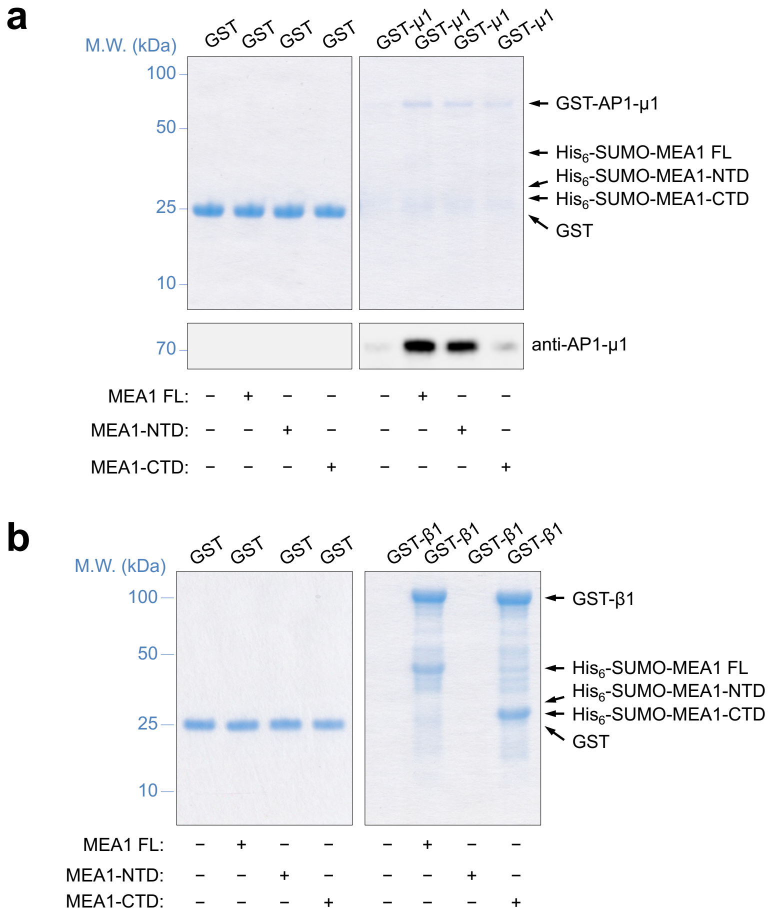

Figure S7 MEA1 uses its NTD and CTD to interact with the μ1 and β1 subunits of AP1, respectively. (a, b) Representative Coomassie blue-stained gels and immunoblots showing MEA1:AP1 subunit interactions in GST pull-down assays using a setup similar to Figure 5d. GST and GST-tagged FL μ1 (a) or the core domain of β1 (b) were individually co-expressed with His_6-SUMO-tagged MEA1 (FL, NTD, or CTD) in *E. coli*. Proteins were isolated using glutathione beads. Related to Figure 5.

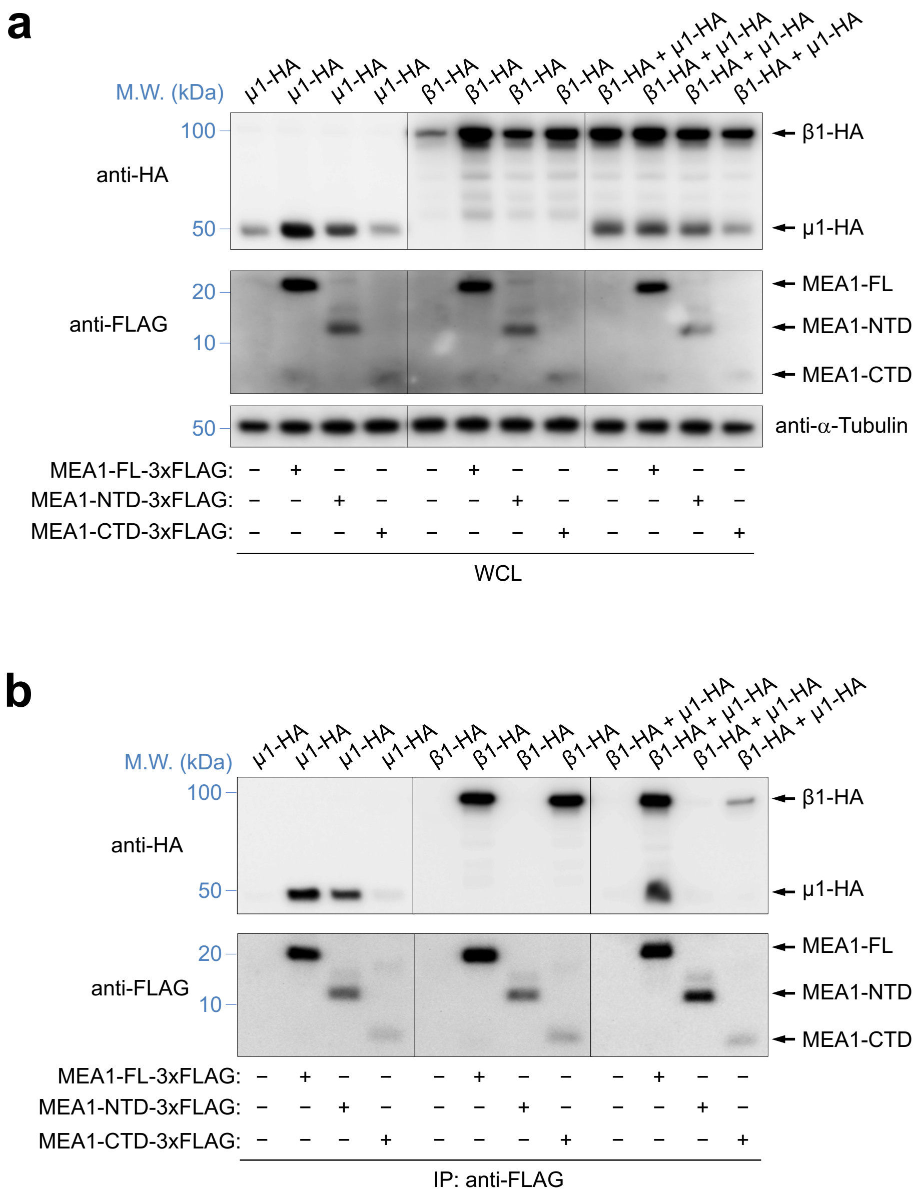

Figure S8. MEA1 possesses two AP1-binding domains. (a) Representative immunoblots showing the expression of the indicated proteins in the WCL of HEK 293T cells. **(b)** Representative immunoblots showing the interaction of 3xFLAG-tagged MEA1 (FL, NTD, or CTD) with HA-tagged AP1 subunits. Plasmids encoding HA-tagged AP1 subunits were transiently expressed in HEK 293T cells with either an empty vector or plasmid encoding 3xFLAG-tagged MEA1 (FL, NTD or CTD). MEA1 was immunoprecipitated using anti-FLAG antibodies and proteins in the immunoprecipitates were detected by immunoblotting. Related to Figure 5.

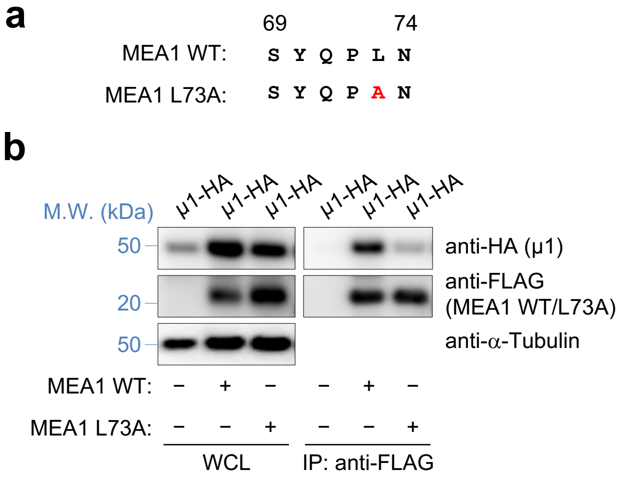

Figure S9. Targeted mutations in the NTD of MEA1 impair µ1 binding. (a) Sequence alignment showing the mutated residue in the mutant MEA1 protein. The mutated residue is shown in red. **(b)** Representative immunoblots showing the binding of µ1 to WT and mutant MEA1 proteins. The co-IP experiments were conducted as in Figure 1d-e. Related to Figure 5.

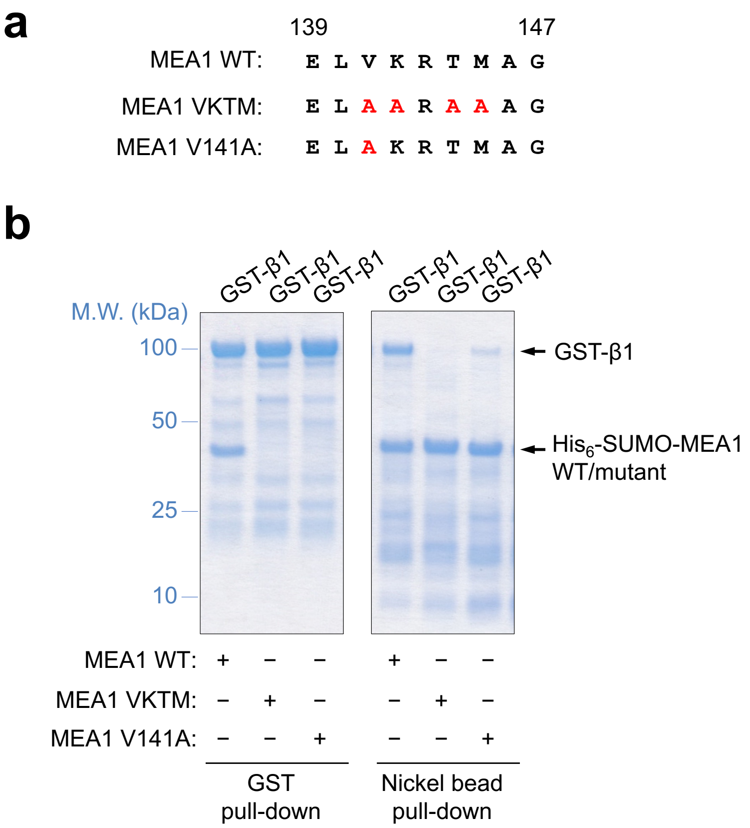

Figure S10. Targeted mutations in the CTD of MEA1 impair β1 binding. (a) Sequence alignment showing the mutated residues in the mutant MEA1 proteins. Mutated residues are shown in red. **(b)** Representative Coomassie blue-stained gels showing the binding of β1 to WT and mutant MEA1 proteins in pull-down assays using a setup similar to Figure 4d-e. The GST and nickel bead pull-down experiments were conducted as in Figure 4d-e. Related to Figure 5.

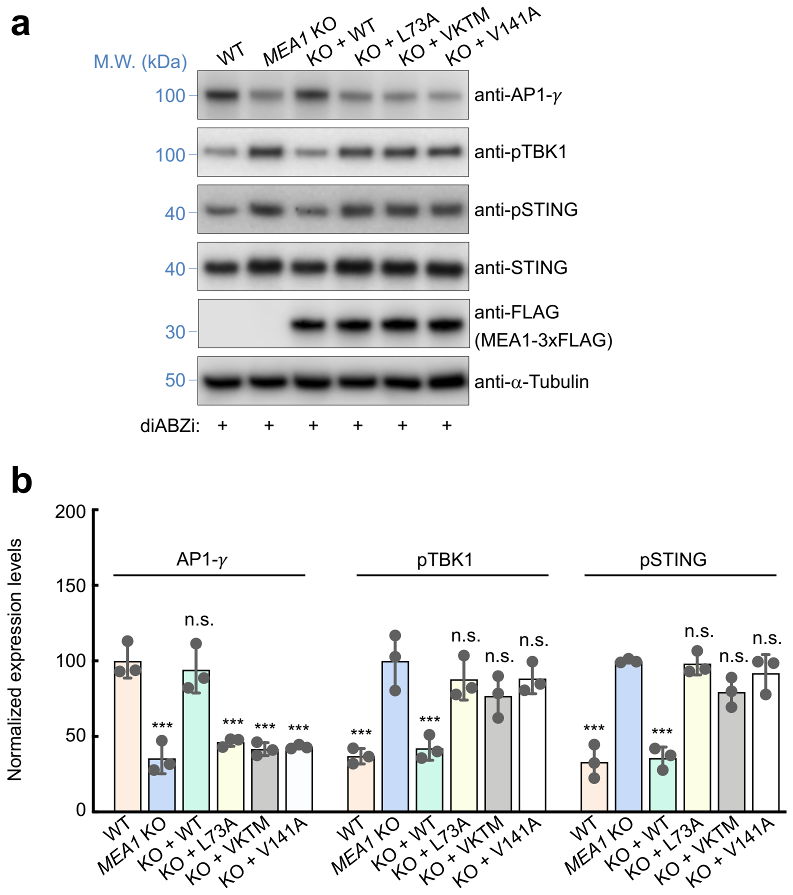

Figure S11. MEA1 function is disrupted by mutations in its AP1-binding sites. (a) Representative immunoblots showing the expression of the indicated proteins in the indicated RPE1 cell lines. **(b)** Quantification of protein expression based on immunoblots in panel a. For γ, data normalization was performed by setting the mean value of WT data points as 100% and normalizing all data points, including WT, to that value. For pTBK1 and pSTING, data normalization was performed by setting the mean value of *MEA1* KO data points as 100% and normalizing all data points accordingly. Data are presented as mean ± SD from three biological replicates. *** $P < 0.001$; n.s., $P > 0.05$ (two-way ANOVA). Related to Figure 5.

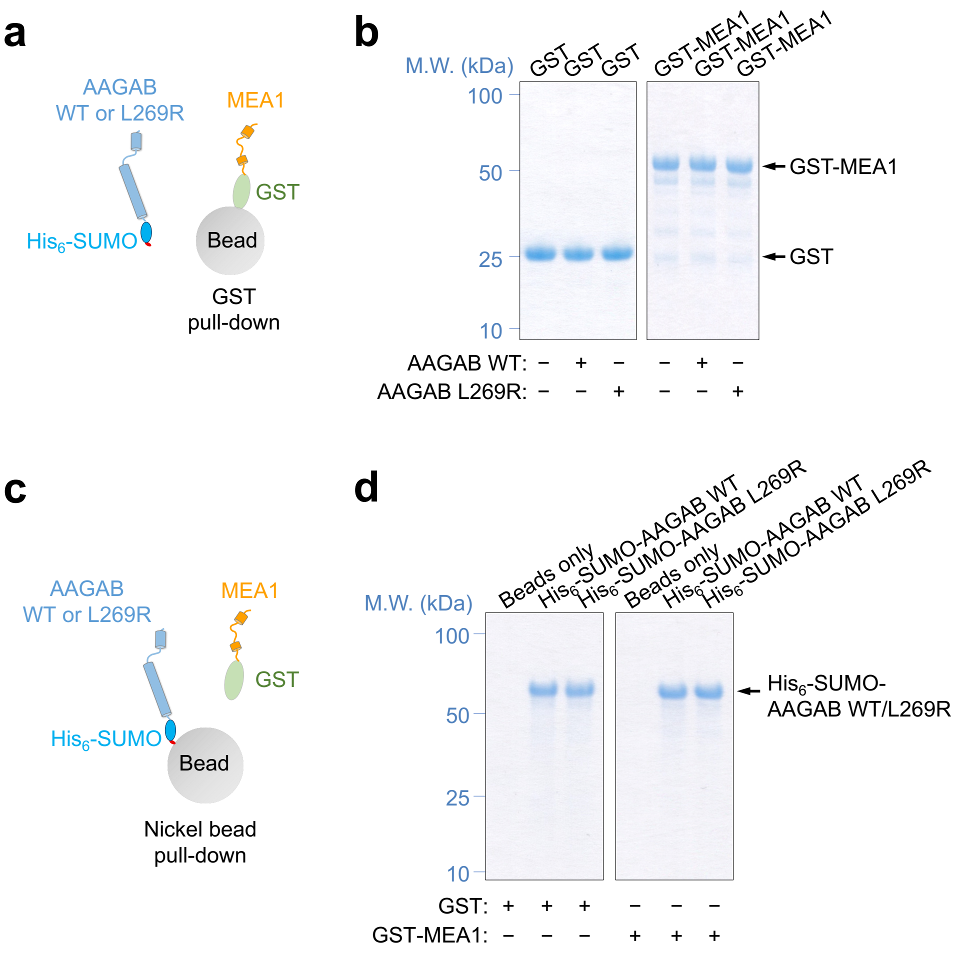

Figure S12. MEA1 does not directly bind to AAGAB. (a) Diagram of the GST pull-down assay detecting the interaction of His_6-SUMO-tagged AAGAB to GST-tagged MEA1 bound to glutathione beads. His_6-SUMO-tagged AAGAB (WT or L269R mutant) was co-expressed with GST or GST-tagged MEA1 in *E. coli*. Proteins were isolated from *E. coli* lysates using glutathione beads. **(b)** Representative Coomassie blue-stained gels showing the results of the GST pull-down assay depicted in panel a. **(c)** Diagram of the nickel bead pull-down assay detecting the interaction of GST-tagged MEA1 with His_6-SUMO-tagged AAGAB (WT or L269R mutant) bound to nickel beads. The L269R mutation disrupts AAGAB homodimerization, resulting in monomeric AAGAB. His_6-SUMO-tagged AAGAB (WT or L269R mutant) and GST-tagged MEA1 were co-expressed in *E. coli* as in a-b. Proteins were isolated using nickel beads recognizing a His_6 tag on SUMO-AAGAB. **(d)** Representative Coomassie blue-stained gels showing the results of the nickel bead pull-down assay depicted in panel c. Related to Figure 6.

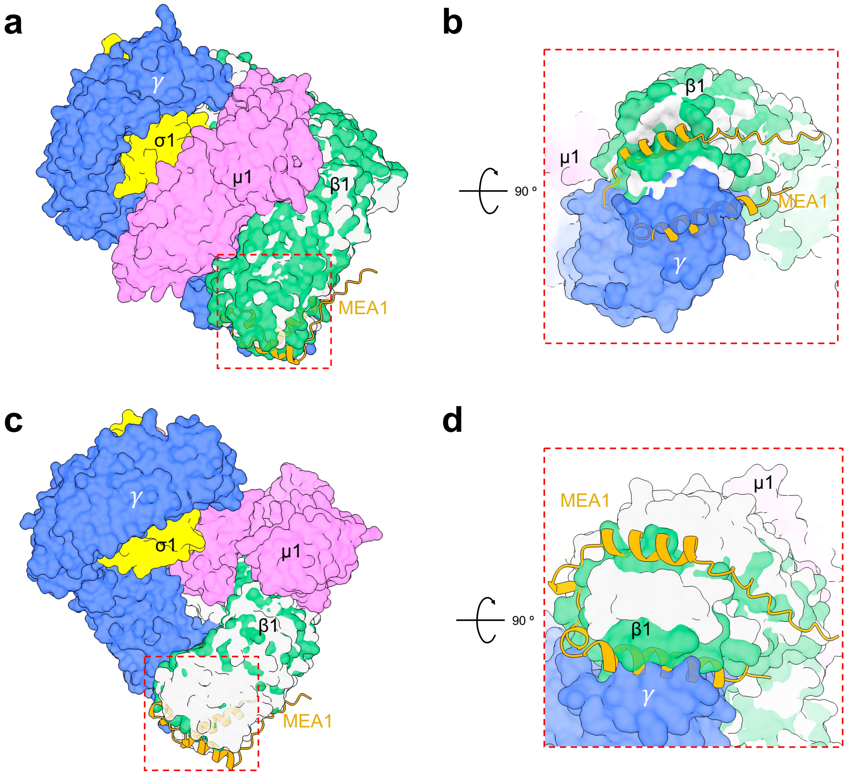

Figure S13 MEA1 binding is incompatible with β1:γ association during AP1 adaptor formation. (a) Superposition of the AlphaFold-predicted structure of the MEA1:β1:µ1 complex (depicted in Figure S4) with the crystal structure of the closed full AP1 adaptor (PDB: 1W63)[1]. **(b)** Enlarged view showing MEA1 occupying the γ-binding site on β1, corresponding to the dashed box in panel a, shown with the indicated rotation. **(c)** Superposition of the AlphaFold-predicted structure of the MEA1:β1:µ1 complex with the crystal structure of the open full AP1 adaptor (PDB: 4HMY)[2]. **(d)** Enlarged view showing MEA1 occupying the γ-binding site on β1, corresponding to the dashed box in panel c, shown with the indicated rotation. MEA1 residues 121-185 are shown; the predicted β1 structure is shown in gray, and the predicted µ1 structure is omitted for clarity. Related to Figure 6.

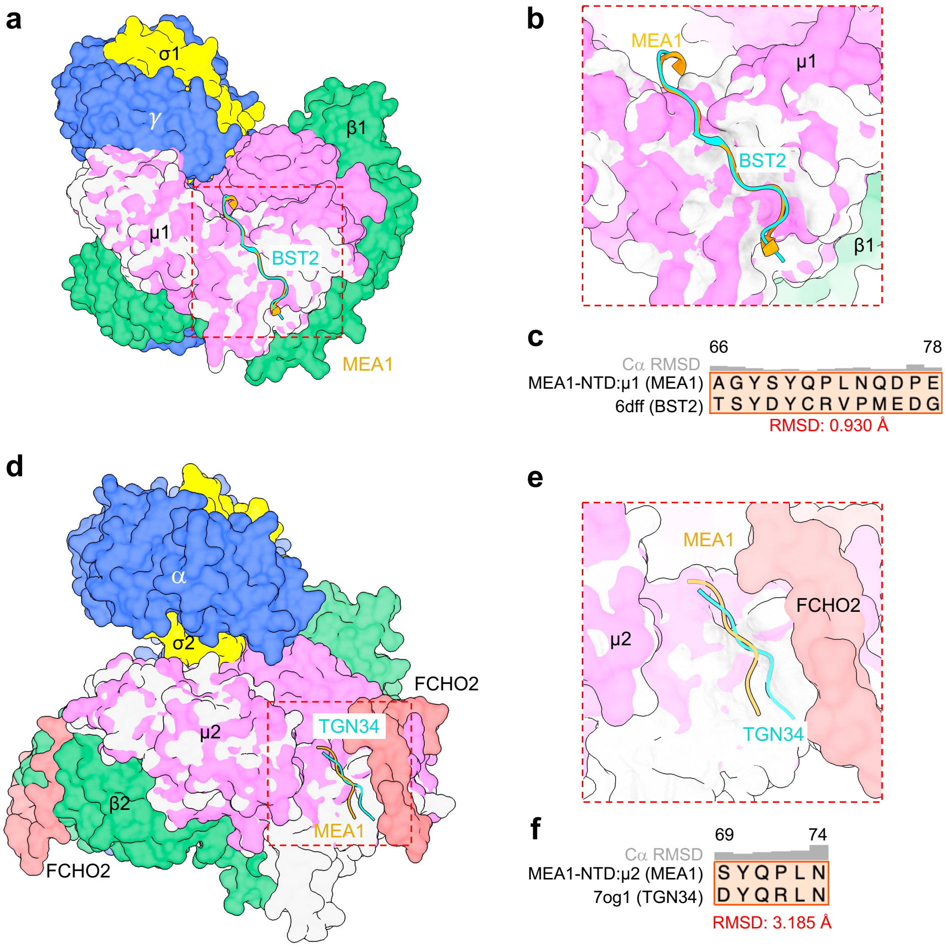

Figure S14. MEA1 occupies the cargo-binding site on μ1 and μ2. (a) Superposition of the AlphaFold-predicted structure of the MEA1-NTD:μ1 complex with the crystal structure of the cargo (BST2)-bound full AP1 adaptor (PDB: 6dff; PMID: 30053425; ARF1 and NEF omitted for clarity). **(b)** Enlarged view of MEA1 bound to the tyrosine-based cargo-binding site on μ1, corresponding to the dashed box in panel a. **(c)** Structural comparison of MEA1 (a.a. 66-78) and BST2 peptide in the indicated protein complexes. **(d)** Superposition of the AlphaFold-predicted structure of the MEA1-NTD:μ2 complex with the crystal structure of the cargo (TGN34)-bound full AP2 adaptor (PDB: 7og1; PMID: 35486718). The CIF file of the predicted structure is included in Supplementary Data 6. **(e)** Enlarged view of MEA1 bound to the tyrosine-based cargo-binding site on μ2, corresponding to the dashed box in panel d. **(f)** Structural comparison of MEA1 (a.a. 69-74) and the TGN34 peptide within the indicated protein complexes. Related to Discussion.

References

1. Heldwein EE, Macia E, Wang J, Yin HL, Kirchhausen T, Harrison SC. Crystal structure of the clathrin adaptor protein 1 core. *Proc Natl Acad Sci U S A* **101**, 14108-14113 (2004).
2. Ren X, Farias GG, Canagarajah BJ, Bonifacino JS, Hurley JH. Structural basis for recruitment and activation of the AP-1 clathrin adaptor complex by Arf1. *Cell* **152**, 755-767 (2013).
