# Supplementary material for: Regulation of AP1 adaptor assembly by the bi-handed chaperone MEA1
Source: Nat Commun. 2026 Jan 20;17:1876. doi: 10.1038/s41467-026-68662-3 (PMC12923701; doi:10.1038/s41467-026-68662-3)
Supplement: Supplementary file 2 — Descriptions of Additional Supplementary Files [file 41467_2026_68662_MOESM2_ESM.pdf]

Descriptions of Additional Supplementary Files

- **Supplementary Data 1**
  Interactomes of human AP1 subunits.
  Related to **Figure 1**.
- **Supplementary Data 2**
  AlphaFold-predicted structure of the MEA1:β1:μ1 trimer
  (CIF file; ModelArchive ID: ma-0gpn5).
  Related to **Supplementary Figure 4**.
- **Supplementary Data 3**
  AlphaFold-predicted structure of the MEA1-NTD:μ1 dimer
  (CIF file; ModelArchive ID: ma-yfe7m).
  Related to **Figure 5**.
- **Supplementary Data 4**
  AlphaFold-predicted structure of the MEA1-CTD:β1 dimer
  (CIF file; ModelArchive ID: ma-h28fe).
  Related to **Figure 5**.
- **Supplementary Data 5**
  AlphaFold-predicted structure of the MEA1:β2:μ2 trimer
  (CIF file; ModelArchive ID: ma-9zsqb).
  Related to **Supplementary Figure 6**.
- **Supplementary Data 6**
  AlphaFold-predicted structure of the MEA1-NTD:μ2 dimer
  (CIF file; ModelArchive ID: ma-uzxwb).
  Related to **Discussion**.
