# Supplementary material for: Regulation of AP1 adaptor assembly by the bi-handed chaperone MEA1
Source: Nat Commun. 2026 Jan 20;17:1876. doi: 10.1038/s41467-026-68662-3 (PMC12923701; doi:10.1038/s41467-026-68662-3)
Supplement: Supplementary file 9 — Reporting Summary [file 41467_2026_68662_MOESM9_ESM.pdf]

nature portfolio

Corresponding author(s): Qian Yin, Jingshi Shen

Last updated by author(s): Nov 24, 2025

Reporting Summary

Nature Portfolio wishes to improve the reproducibility of the work that we publish. This form provides structure for consistency and transparency in reporting. For further information on Nature Portfolio policies, see our Editorial Policies and the Editorial Policy Checklist.

Statistics

For all statistical analyses, confirm that the following items are present in the figure legend, table legend, main text, or Methods section.

n/a	Confirmed	
☐	☒	The exact sample size (*n*) for each experimental group/condition, given as a discrete number and unit of measurement
☐	☒	A statement on whether measurements were taken from distinct samples or whether the same sample was measured repeatedly
☐	☒	The statistical test(s) used AND whether they are one- or two-sided<br>*Only common tests should be described solely by name; describe more complex techniques in the Methods section.*
☒	☐	A description of all covariates tested
☒	☐	A description of any assumptions or corrections, such as tests of normality and adjustment for multiple comparisons
☐	☒	A full description of the statistical parameters including central tendency (e.g. means) or other basic estimates (e.g. regression coefficient) AND variation (e.g. standard deviation) or associated estimates of uncertainty (e.g. confidence intervals)
☐	☒	For null hypothesis testing, the test statistic (e.g. *F*, *t*, *r*) with confidence intervals, effect sizes, degrees of freedom and *P* value noted<br>*Give P values as exact values whenever suitable.*
☒	☐	For Bayesian analysis, information on the choice of priors and Markov chain Monte Carlo settings
☒	☐	For hierarchical and complex designs, identification of the appropriate level for tests and full reporting of outcomes
☐	☒	Estimates of effect sizes (e.g. Cohen's *d*, Pearson's *r*), indicating how they were calculated

Our web collection on statistics for biologists contains articles on many of the points above.

Software and code

Policy information about availability of computer code

Data collection	The Western blots data: Bio-Rad ChemiDoc Imager<br>The Flow data: Beckman Coulter CyAn-ADP analyzer<br>Phylogenetic data: Phylogeny.fr and Uniprot<br>Structure data: AlphaFold3<br>Images data: Nikon A100
Data analysis	AlphaFold3, UCSF ChimeraX-1.7, ImageJ-2.14, Flowjo-10.8, and Graphpad Prism-9.0

For manuscripts utilizing custom algorithms or software that are central to the research but not yet described in published literature, software must be made available to editors and reviewers. We strongly encourage code deposition in a community repository (e.g. GitHub). See the Nature Portfolio guidelines for submitting code & software for further information.

Data

Policy information about availability of data

All manuscripts must include a data availability statement. This statement should provide the following information, where applicable:

- Accession codes, unique identifiers, or web links for publicly available datasets
- A description of any restrictions on data availability
- For clinical datasets or third party data, please ensure that the statement adheres to our policy

Source data are provided in this paper. The in-silico screening data are available in Table 1. AlphaFold3-predicted structural models are included as Supplementary Data. Additional data are available from the corresponding authors upon reasonable request.

Research involving human participants, their data, or biological material

Policy information about studies with human participants or human data. See also policy information about sex, gender (identity/presentation), and sexual orientation and race, ethnicity and racism.

Reporting on sex and gender	not applicable
Reporting on race, ethnicity, or other socially relevant groupings	not applicable
Population characteristics	not applicable
Recruitment	not applicable
Ethics oversight	not applicable

Note that full information on the approval of the study protocol must also be provided in the manuscript.

Field-specific reporting

Please select the one below that is the best fit for your research. If you are not sure, read the appropriate sections before making your selection.

☒ Life sciences ☐ Behavioural & social sciences ☐ Ecological, evolutionary & environmental sciences

For a reference copy of the document with all sections, see nature.com/documents/nr-reporting-summary-flat.pdf

Life sciences study design

All studies must disclose on these points even when the disclosure is negative.

Sample size	The sample sizes are clearly detailed in the figure legends.
Data exclusions	No data were excluded from the analyses.
Replication	All biological experiments were performed at least three times to ensure reproducibility. Western blot data were validated using a minimum of three independent samples.
Randomization	The randomization is clearly detailed in the figure legends.
Blinding	No blinding was used.

Reporting for specific materials, systems and methods

We require information from authors about some types of materials, experimental systems and methods used in many studies. Here, indicate whether each material, system or method listed is relevant to your study. If you are not sure if a list item applies to your research, read the appropriate section before selecting a response.

Materials & experimental systems

n/a	Involved in the study
☐	☒ Antibodies
☐	☒ Eukaryotic cell lines
☒	☐ Palaeontology and archaeology
☒	☐ Animals and other organisms
☒	☐ Clinical data
☒	☐ Dual use research of concern
☒	☐ Plants

Methods

n/a	Involved in the study
☒	☐ ChIP-seq
☐	☒ Flow cytometry
☒	☐ MRI-based neuroimaging

Antibodies

Antibodies used

Primary antibodies used in immunoblotting included polyclonal anti-MEA1 antibodies (Bethyl Laboratories, #A305-779A, RRID: AB_2891676), polyclonal anti-STING antibodies (Proteintech, #19851-1-AP, RRID: AB_10665370), monoclonal anti-pSTING antibodies (Ser366, Cell Signaling Technology #19781, RRID: AB_2737062), monoclonal anti-pTBK1 antibodies (Ser172, Cell Signaling Technology, #5483, RRID:AB_10693472), polyclonal anti-clathrin heavy chain (CHC) antibodies (Cell Signaling Technology, #2410, RRID: AB_2083156), polyclonal anti-γ antibodies (Bethyl Laboratories, #A304-771A, RRID: AB_2620966), polyclonal anti-beta1-adaptin antibodies (ProteinTech, #16932-1-AP, RRID: AB_2274034), polyclonal anti-μ1 antibodies (MyBioSource, #MBS712215), polyclonal anti-σ1 antibodies (Bethyl Laboratories, #A305-396A, RRID: AB_2631787), monoclonal anti-α-adaptin antibodies (BD Biosciences, #610502, RRID: AB_397868), polyclonal anti-β2 antibodies (Bethyl Laboratories, #A304-719A, RRID: AB_2620914), monoclonal anti-μ2 antibodies (BD Biosciences, #611350), polyclonal anti-σ2 antibodies (Abcam, #ab128950, RRID: AB_11140842), monoclonal anti-δ antibodies (DSHB, #SA4), monoclonal anti-AP4E1-antibodies (BD Biosciences, #612018), monoclonal anti-AP5Z1 antibodies (Thermo Fisher Scientific, #66533-1-IG), anti-FLAG M2 antibodies (Sigma-Aldrich, #F1804, RRID: AB_262044), ALFA-tagged MEA1 was labeled using mScarlet-anti-ALFA nanobodies, anti- -adaptin antibodies (Sigma, A4200, RRID: AB_476720), and monoclonal anti-α-tubulin antibodies (DSHB, #12G10, RRID: AB_1210456). Secondary antibodies used in immunoblotting included horseradish peroxidase (HRP)-conjugated anti-rabbit antibodies (Sigma-Aldrich, #A6154, RRID: AB_258284), and HRP-conjugated anti-mouse antibodies (Sigma-Aldrich, #A6782, RRID: AB_258315). FLAG-tagged proteins were directly detected using HRP-conjugated anti-FLAG M2 antibodies (Sigma-Aldrich, #A8592, RRID: AB_439702). HA-tagged proteins were directly detected using HRP-conjugated anti-HA antibodies (Roche, #12013819001, RRID: AB_390917). Secondary antibodies used in flow cytometry and imaging included Alexa Fluor 488-conjugated secondary antibodies (Thermo Fisher Scientific, #A11008, RRID: AB_142165), anti-mouse Alexa Fluor 568-conjugated secondary antibodies (Thermo Fisher Scientific, #A11004, RRID: AB_2534072), anti-rabbit Alexa Fluor 568-conjugated secondary antibodies (Thermo Fisher Scientific, #A11011, RRID: AB_143157). The plasma membrane was stained with CF405-conjugated Concanavalin A (Biotum, #29074).

Validation

These are commercial antibodies that have been widely used and reported in many publications.

Eukaryotic cell lines

Policy information about cell lines and Sex and Gender in Research

Cell line source(s)

HeLa, HEK293T, RPE1

Authentication

All cell lines were purchased from ATCC

Mycoplasma contamination

Cells were routinely tested for mycoplasma contamination with Hoechst 33342 and routinely maintained in Normocin (Invivogen)

Commonly misidentified lines (See ICLAC register)

Not applicable

Plants

Seed stocks

Not applicable

Novel plant genotypes

Not applicable

Authentication

Not applicable

Flow Cytometry

Plots

Confirm that:

☐ The axis labels state the marker and fluorochrome used (e.g. CD4-FITC).

☐ The axis scales are clearly visible. Include numbers along axes only for bottom left plot of group (a 'group' is an analysis of identical markers).

☐ All plots are contour plots with outliers or pseudocolor plots.

☒ A numerical value for number of cells or percentage (with statistics) is provided.

Methodology

Sample preparation	HEK 293T cells, HeLa cells, and RPE1 cells were cultured in Dulbecco's Modified Eagle Medium (DMEM) supplemented with 10% Fetal Bovine Serum (FBS, Sigma, #F0926) and penicillin/streptomycin (Corning, #130-002-CI). For flow cytometry, cells were washed with KRH buffer (12 mM HEPES, pH 7.0, 121 mM NaCl, 4.9 mM KCl, 1.2 mM MgSO4, and 0.33 mM CaCl2) and blocked with KRH buffer containing 5% FBS at 4 ºC. To measure surface FOLR1, unpermeabilized cells were stained using PE-conjugated anti-FOLR1 antibodies (BioLegend, #908304, RRID: AB_2629795). For immunostaining and imaging, cells grown on coverslips were fixed using 4% paraformaldehyde (PFA) and permeabilized in PBS containing 5% FBS and 0.2% saponin, then stained with indicated antibodies. For immunoblotting and immunoprecipitation, cells grown in 24-well plates were lysed in a SDS protein sample buffer (80 mM Tris, pH 6.8, 2% SDS, 10% glycerol, 0.0006% Bromophenol blue, and 0.1 M DTT). The samples were resolved on 8% Bis-Tris SDS-PAGE, transferred to PVDF membranes, and probed using indicated antibodies. For cell fractionation and CCV isolation, . Cells grown on 10-cm dishes were either untreated or treated with 2.5 µM diABZI (Selleckchem, #S8796) for 2 hours. Cells were washed with PBS and harvested into Buffer A (0.1 M MES, pH 6.5, 0.2 mM EGTA, and 0.5 mM $MgCl_2$). Cell lysis was performed by repeated pipetting (>25 times) using a 5 mL syringe fitted with a 22-gauge needle, and CCV were extracted via multiple rounds of ultra-centrifugation. Recombinant proteins were expressed in BL21 (DE3) E. coli (Stratagene, #230132) and purified using established procedures.
Instrument	Beckman Coulter CyAn ADP analyzer, Beckman ultra-centrifuge LM-80, Nikon A100
Software	ImageJ-2.14, Flowjo-10.8, and Graphpad Prism-9.0
Cell population abundance	Samples were not sorted; whole cells were analyzed by flow cytometry after dissociation.
Gating strategy	Cells were gated based on a clear population using FSC/SSC setting of CyAn, and ~5,000 cells were collected for analysis.

☐ Tick this box to confirm that a figure exemplifying the gating strategy is provided in the Supplementary Information.
