# Supplementary material for: Regulation of AP1 adaptor assembly by the bi-handed chaperone MEA1
Source: Nat Commun. 2026 Jan 20;17:1876. doi: 10.1038/s41467-026-68662-3 (PMC12923701; doi:10.1038/s41467-026-68662-3)
Supplement: Supplementary file 11 — Source Data [file 41467_2026_68662_MOESM11_ESM.zip › Source Data full blots.pptx]

## Slide 1
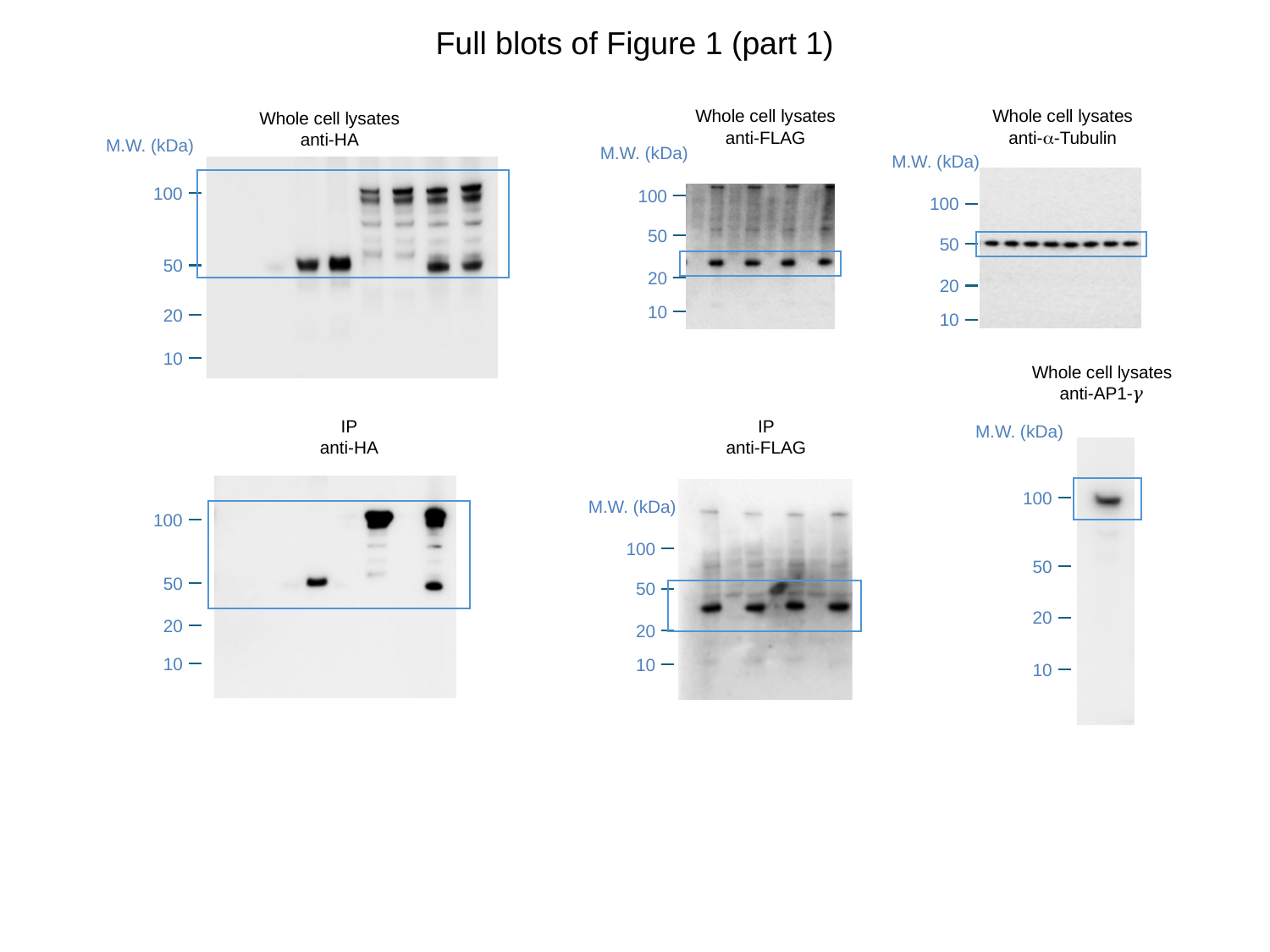

Full blots of Figure 1 (part 1)
Whole cell lysates
anti-FLAG
Whole cell lysates
anti-α-Tubulin
Whole cell lysates
anti-HA
M.W. (kDa)
M.W. (kDa)
M.W. (kDa)
100
100
100
50
50
50
20
20
10
20
10
10
Whole cell lysates
anti-AP1-𝛾
IP
anti-HA
IP
anti-FLAG
M.W. (kDa)
100
M.W. (kDa)
100
100
50
50
50
20
20
20
10
10
10

## Slide 2
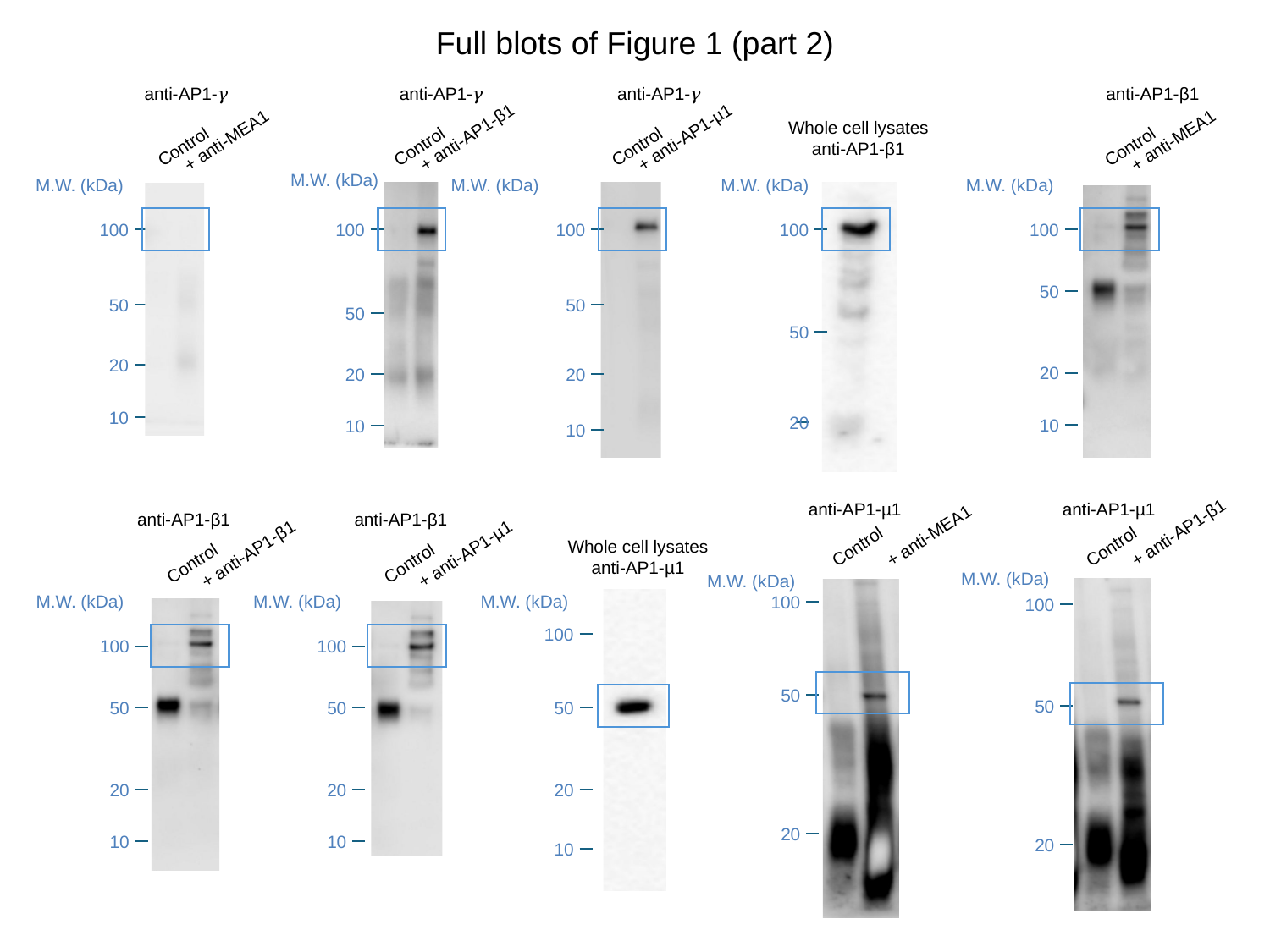

Full blots of Figure 1 (part 2)
anti-AP1-𝛾
anti-AP1-𝛾
anti-AP1-𝛾
anti-AP1-β1
Whole cell lysates
anti-AP1-β1
+ anti-AP1-β1
+ anti-AP1-µ1
+ anti-MEA1
Control
Control
Control
Control
+ anti-MEA1
M.W. (kDa)
M.W. (kDa)
M.W. (kDa)
M.W. (kDa)
M.W. (kDa)
100
100
100
100
100
50
50
50
50
50
20
20
20
20
10
20
10
10
10
anti-AP1-µ1
anti-AP1-µ1
anti-AP1-β1
anti-AP1-β1
+ anti-AP1-β1
Control
+ anti-MEA1
Control
+ anti-AP1-β1
+ anti-AP1-µ1
Whole cell lysates
anti-AP1-µ1
Control
Control
M.W. (kDa)
M.W. (kDa)
M.W. (kDa)
M.W. (kDa)
M.W. (kDa)
100
100
100
100
100
50
50
50
50
50
20
20
20
20
10
10
20
10

## Slide 3
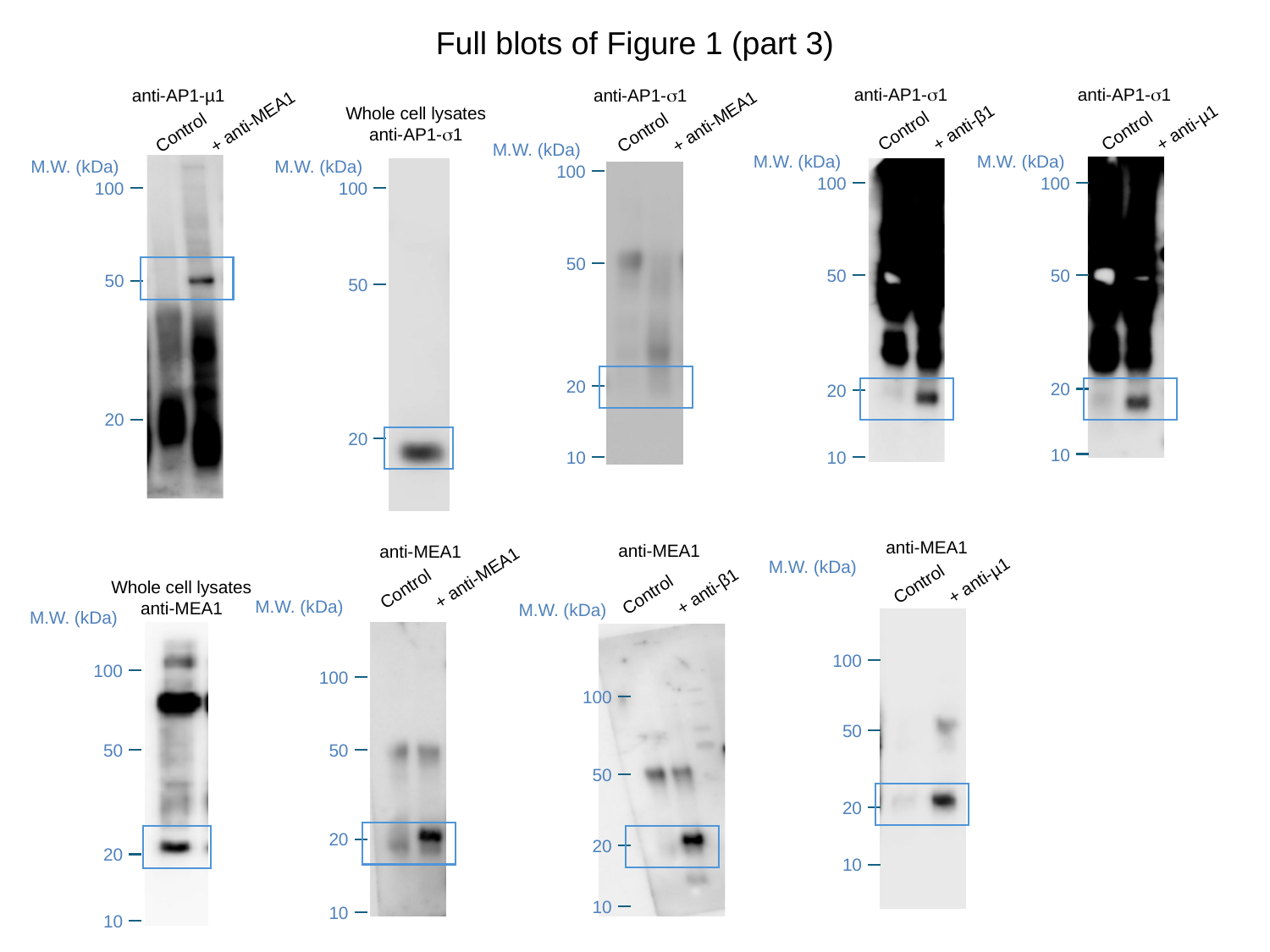

Full blots of Figure 1 (part 3)
anti-AP1-σ1
anti-AP1-σ1
anti-AP1-µ1
anti-AP1-σ1
Whole cell lysates
anti-AP1-σ1
Control
+ anti-β1
Control
+ anti-µ1
Control
+ anti-MEA1
Control
+ anti-MEA1
M.W. (kDa)
M.W. (kDa)
M.W. (kDa)
M.W. (kDa)
M.W. (kDa)
100
100
100
100
100
50
50
50
50
50
20
20
20
20
20
10
10
10
anti-MEA1
anti-MEA1
anti-MEA1
M.W. (kDa)
Control
+ anti-µ1
Control
+ anti-MEA1
Control
+ anti-β1
Whole cell lysates
anti-MEA1
M.W. (kDa)
M.W. (kDa)
M.W. (kDa)
100
100
100
100
50
50
50
50
20
20
20
20
10
10
10
10

## Slide 4
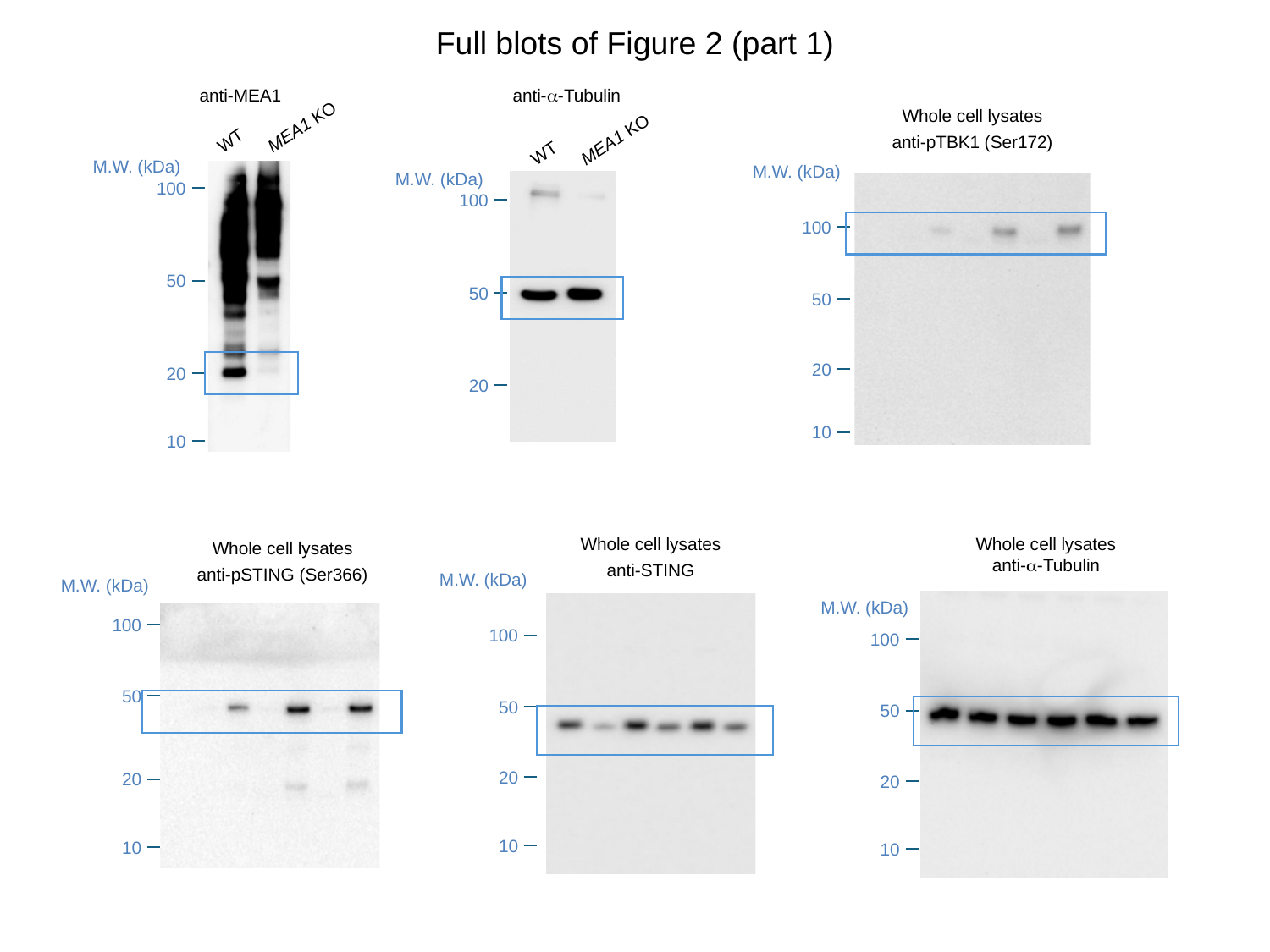

Full blots of Figure 2 (part 1)
anti-MEA1
anti-α-Tubulin
Whole cell lysates
anti-pTBK1 (Ser172)
WT
MEA1 KO
WT
MEA1 KO
M.W. (kDa)
M.W. (kDa)
M.W. (kDa)
100
100
100
50
50
50
20
20
20
10
10
Whole cell lysates
anti-STING
Whole cell lysates
anti-α-Tubulin
Whole cell lysates
anti-pSTING (Ser366)
M.W. (kDa)
M.W. (kDa)
M.W. (kDa)
100
100
100
50
50
50
20
20
20
10
10
10

## Slide 5
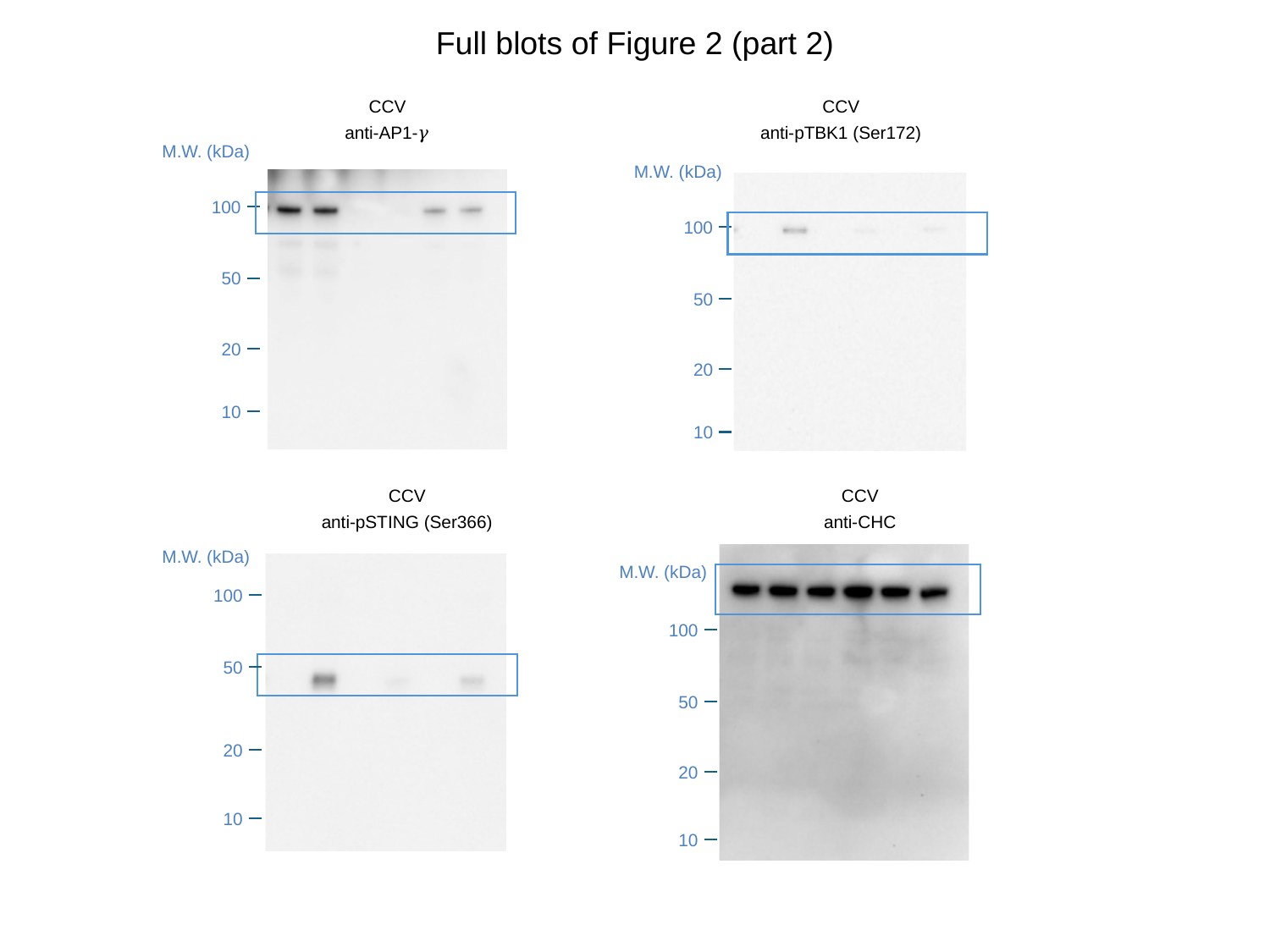

Full blots of Figure 2 (part 2)
CCV
anti-AP1-𝛾
CCV
anti-pTBK1 (Ser172)
M.W. (kDa)
M.W. (kDa)
100
100
50
50
20
20
10
10
CCV
anti-pSTING (Ser366)
CCV
anti-CHC
M.W. (kDa)
M.W. (kDa)
100
100
50
50
20
20
10
10

## Slide 6
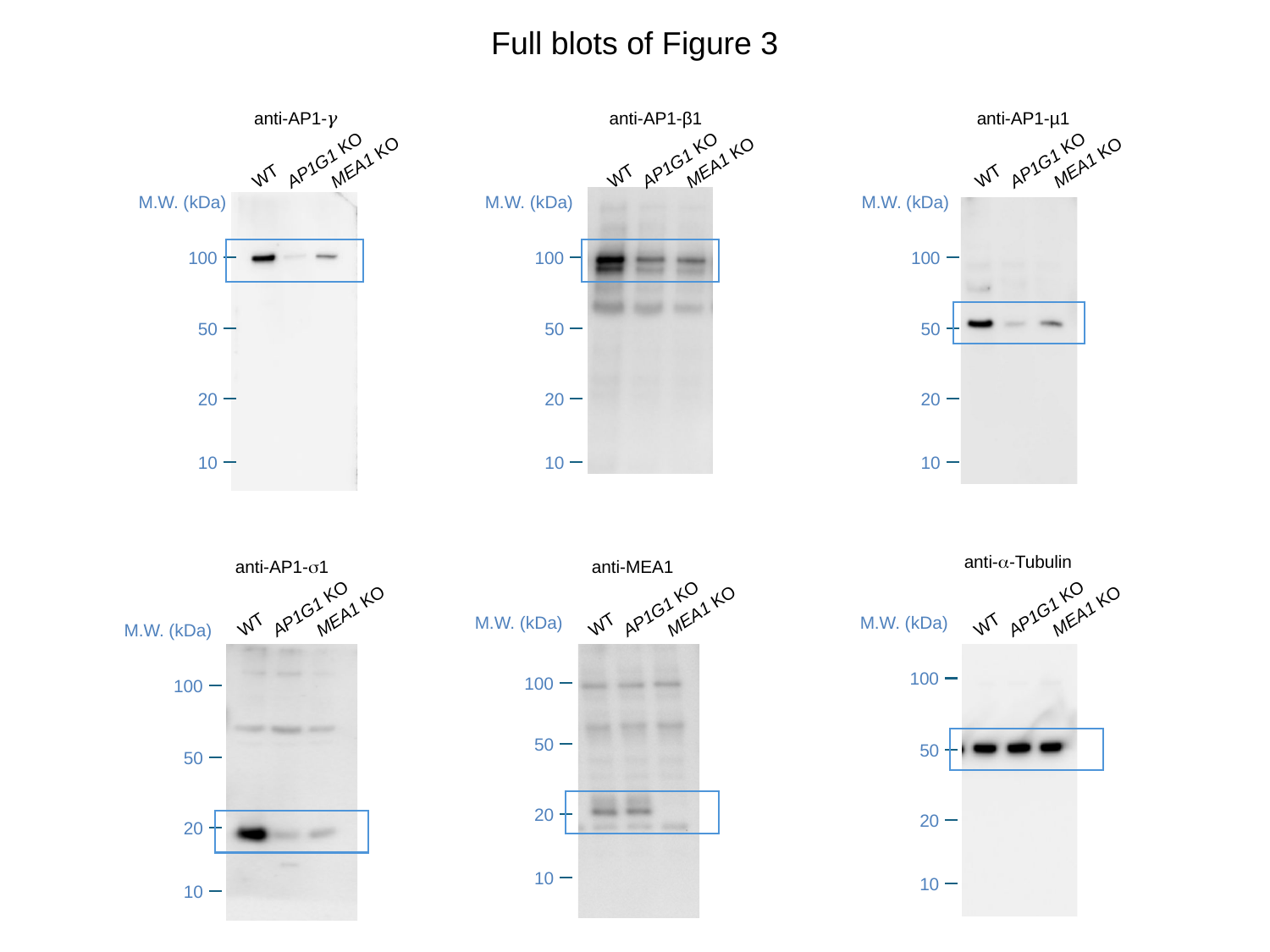

Full blots of Figure 3
anti-AP1-𝛾
anti-AP1-β1
anti-AP1-µ1
WT
WT
WT
AP1G1 KO
AP1G1 KO
AP1G1 KO
MEA1 KO
MEA1 KO
MEA1 KO
M.W. (kDa)
M.W. (kDa)
M.W. (kDa)
100
100
100
50
50
50
20
20
20
10
10
10
anti-AP1-σ1
anti-MEA1
anti-α-Tubulin
WT
WT
WT
AP1G1 KO
AP1G1 KO
AP1G1 KO
MEA1 KO
MEA1 KO
MEA1 KO
M.W. (kDa)
M.W. (kDa)
M.W. (kDa)
100
100
100
50
50
50
20
20
20
10
10
10

## Slide 7
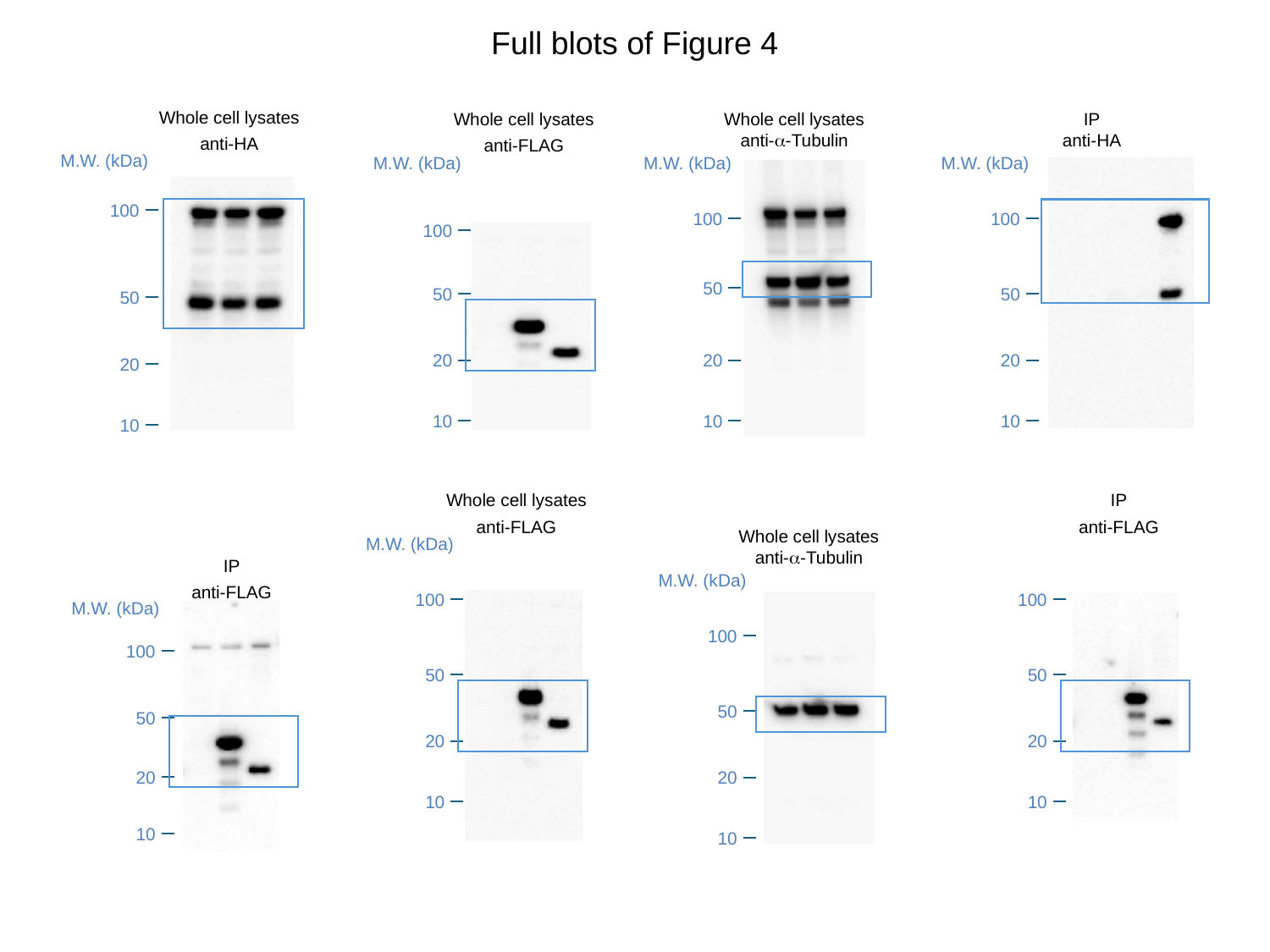

Full blots of Figure 4
Whole cell lysates
anti-HA
Whole cell lysates
anti-FLAG
Whole cell lysates
anti-α-Tubulin
IP
anti-HA
M.W. (kDa)
M.W. (kDa)
M.W. (kDa)
M.W. (kDa)
100
100
100
100
50
50
50
50
20
20
20
20
10
10
10
10
Whole cell lysates
anti-FLAG
IP
anti-FLAG
Whole cell lysates
anti-α-Tubulin
M.W. (kDa)
IP
anti-FLAG
M.W. (kDa)
100
100
M.W. (kDa)
100
100
50
50
50
50
20
20
20
20
10
10
10
10

## Slide 8
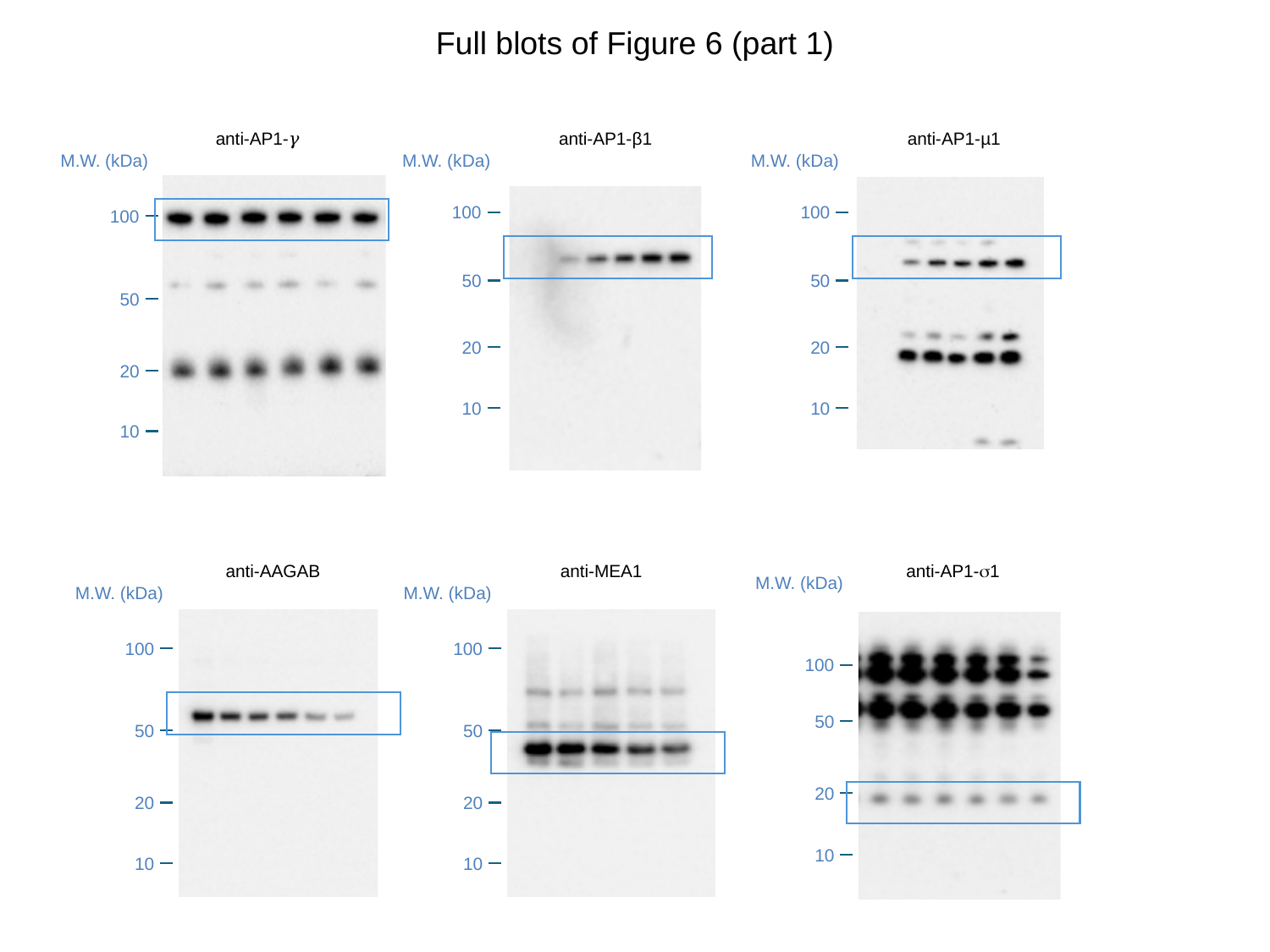

Full blots of Figure 6 (part 1)
anti-AP1-𝛾
anti-AP1-β1
anti-AP1-µ1
M.W. (kDa)
M.W. (kDa)
M.W. (kDa)
100
100
100
50
50
50
20
20
20
10
10
10
anti-AAGAB
anti-MEA1
anti-AP1-σ1
M.W. (kDa)
M.W. (kDa)
M.W. (kDa)
100
100
100
50
50
50
20
20
20
10
10
10

## Slide 9
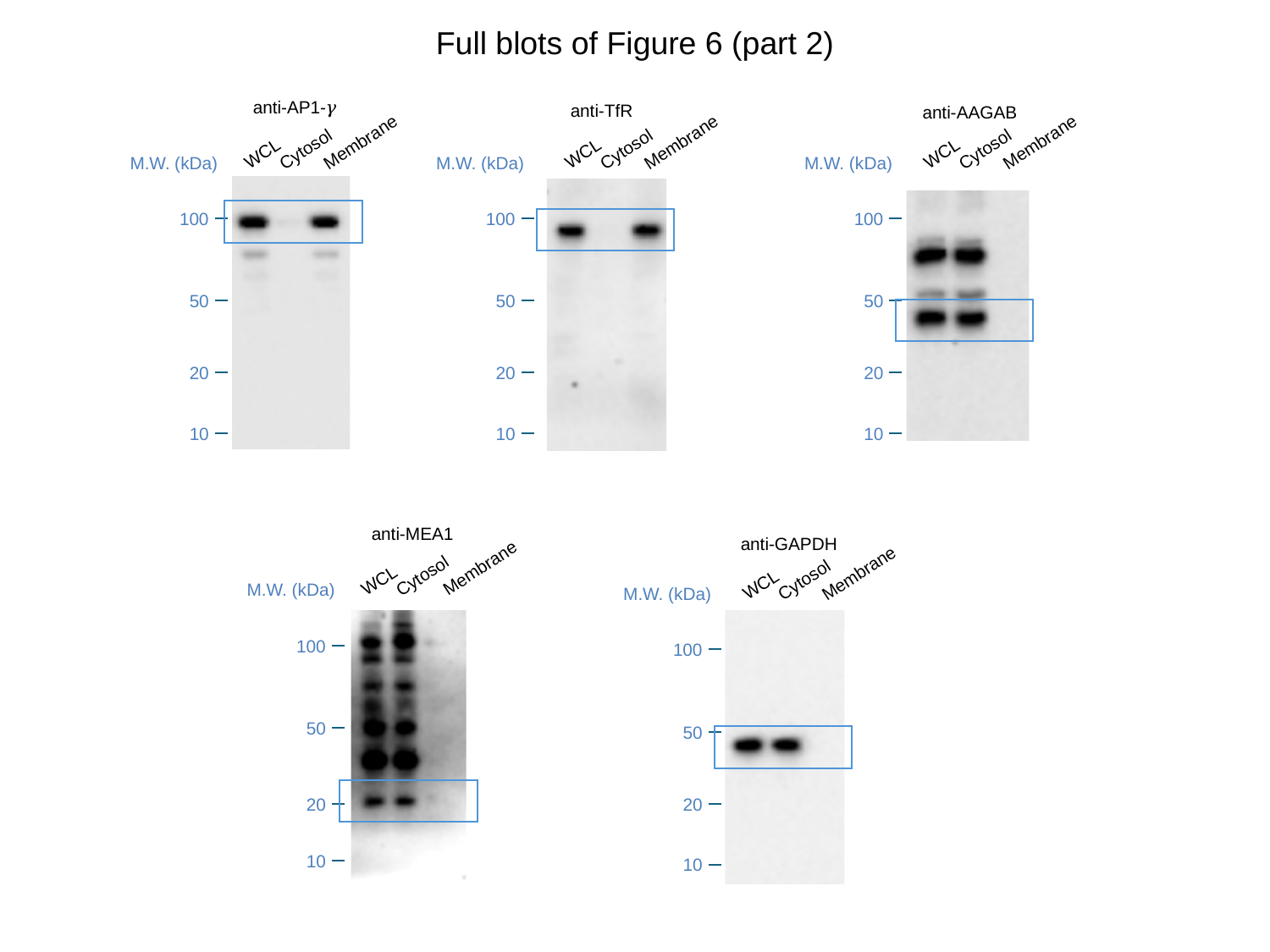

Full blots of Figure 6 (part 2)
anti-AP1-𝛾
anti-TfR
anti-AAGAB
WCL
WCL
WCL
Cytosol
Membrane
Cytosol
Membrane
Cytosol
Membrane
M.W. (kDa)
M.W. (kDa)
M.W. (kDa)
100
100
100
50
50
50
20
20
20
10
10
10
anti-MEA1
anti-GAPDH
Membrane
WCL
Cytosol
WCL
Cytosol
Membrane
M.W. (kDa)
M.W. (kDa)
100
100
50
50
20
20
10
10

## Slide 10
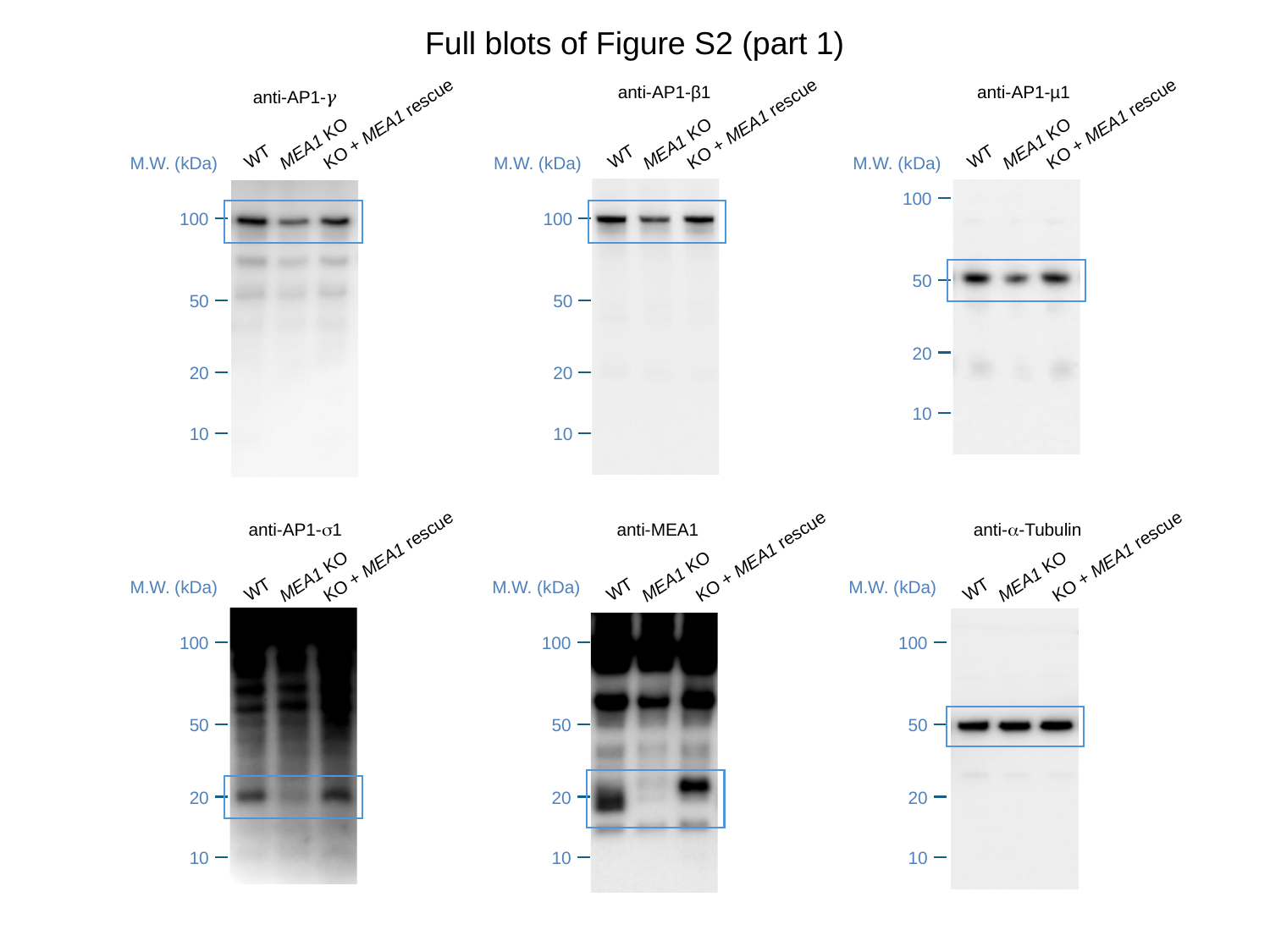

Full blots of Figure S2 (part 1)
anti-AP1-𝛾
anti-AP1-β1
anti-AP1-µ1
KO + MEA1 rescue
KO + MEA1 rescue
KO + MEA1 rescue
WT
WT
WT
MEA1 KO
MEA1 KO
MEA1 KO
M.W. (kDa)
M.W. (kDa)
M.W. (kDa)
100
100
100
50
50
50
20
20
20
10
10
10
anti-AP1-σ1
anti-MEA1
anti-α-Tubulin
KO + MEA1 rescue
KO + MEA1 rescue
KO + MEA1 rescue
WT
WT
WT
MEA1 KO
MEA1 KO
MEA1 KO
M.W. (kDa)
M.W. (kDa)
M.W. (kDa)
100
100
100
50
50
50
20
20
20
10
10
10

## Slide 11
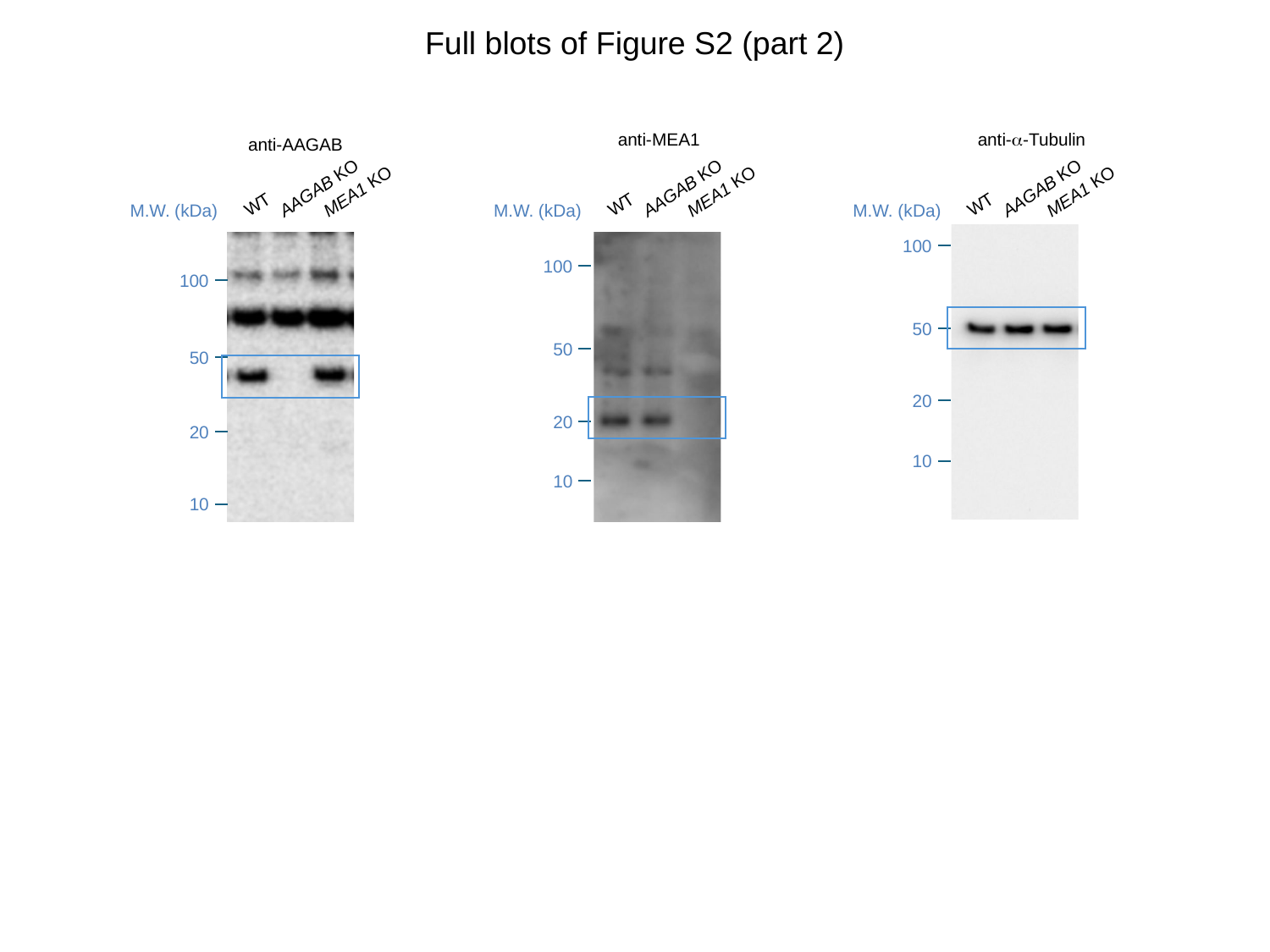

Full blots of Figure S2 (part 2)
anti-AAGAB
anti-MEA1
anti-α-Tubulin
MEA1 KO
MEA1 KO
MEA1 KO
WT
WT
WT
AAGAB KO
AAGAB KO
AAGAB KO
M.W. (kDa)
M.W. (kDa)
M.W. (kDa)
100
100
100
50
50
50
20
20
20
10
10
10

## Slide 12
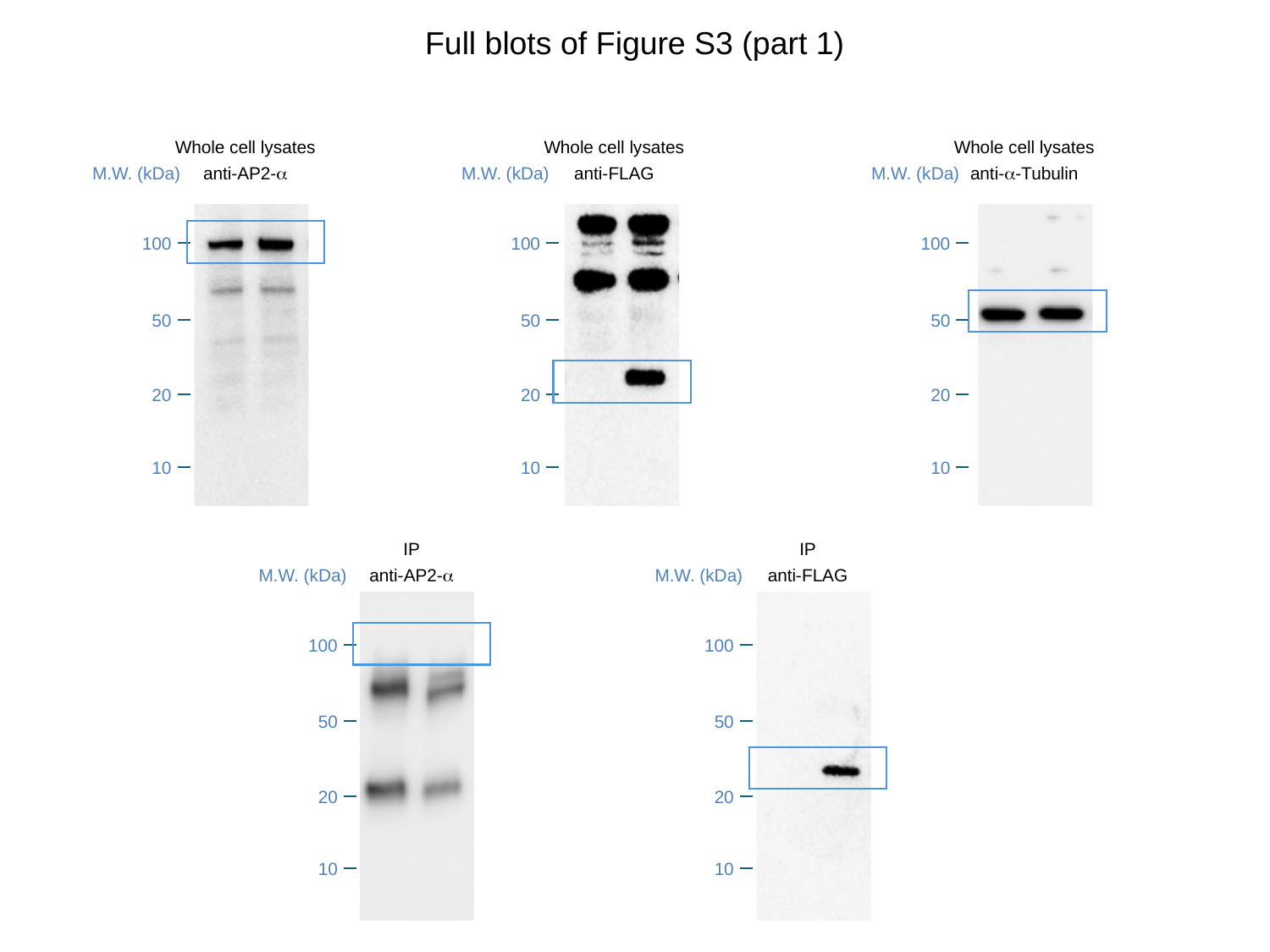

Full blots of Figure S3 (part 1)
Whole cell lysates
anti-AP2-α
Whole cell lysates
anti-FLAG
Whole cell lysates
anti-α-Tubulin
M.W. (kDa)
M.W. (kDa)
M.W. (kDa)
100
100
100
50
50
50
20
20
20
10
10
10
IP
anti-AP2-α
IP
anti-FLAG
M.W. (kDa)
M.W. (kDa)
100
100
50
50
20
20
10
10

## Slide 13
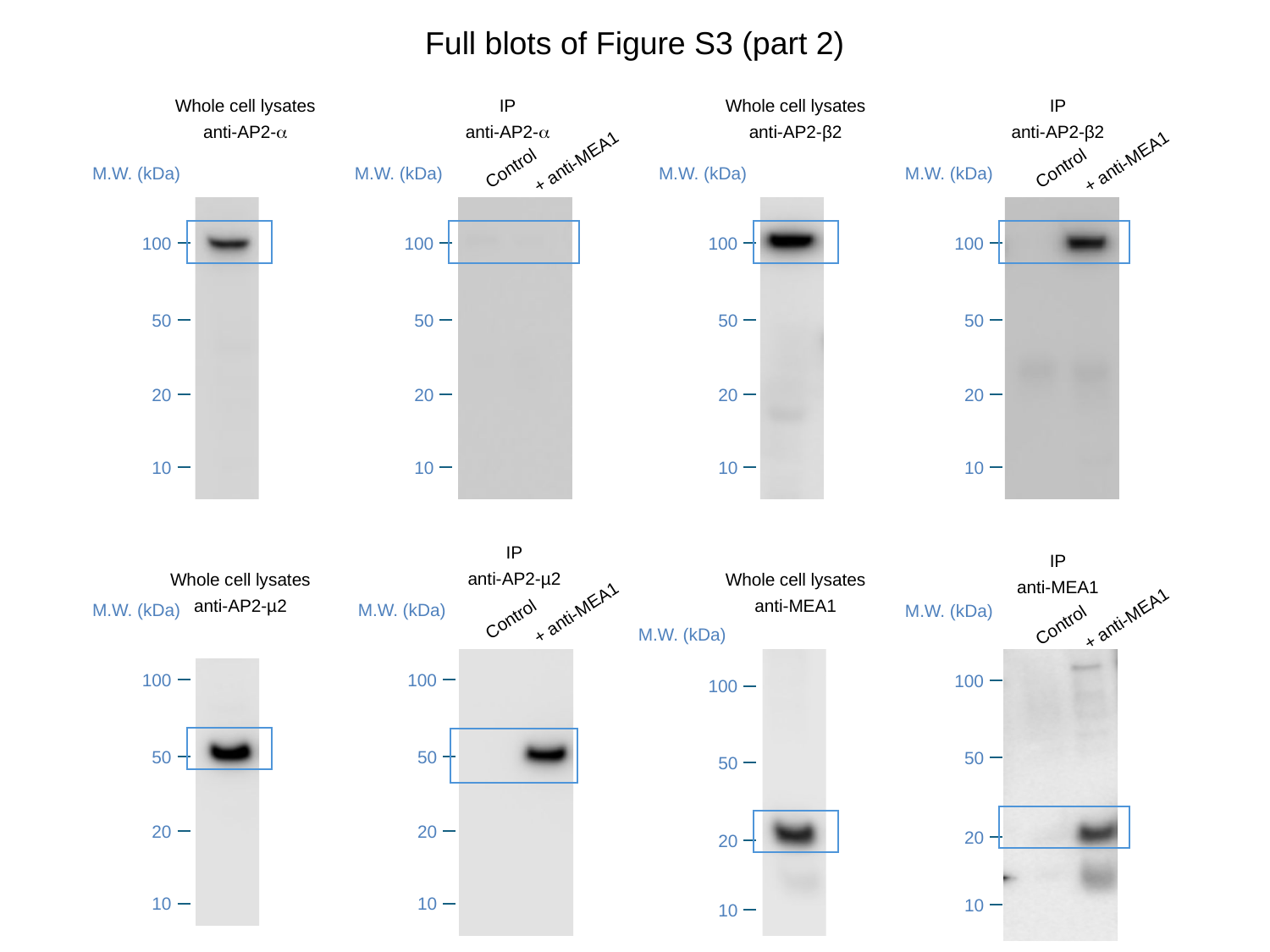

Full blots of Figure S3 (part 2)
Whole cell lysates
anti-AP2-α
IP
anti-AP2-α
Whole cell lysates
anti-AP2-β2
IP
anti-AP2-β2
Control
Control
+ anti-MEA1
+ anti-MEA1
M.W. (kDa)
M.W. (kDa)
M.W. (kDa)
M.W. (kDa)
100
100
100
100
50
50
50
50
20
20
20
20
10
10
10
10
IP
anti-AP2-µ2
IP
anti-MEA1
Whole cell lysates
anti-AP2-µ2
Whole cell lysates
anti-MEA1
Control
M.W. (kDa)
M.W. (kDa)
+ anti-MEA1
M.W. (kDa)
Control
+ anti-MEA1
M.W. (kDa)
100
100
100
100
50
50
50
50
20
20
20
20
10
10
10
10

## Slide 14
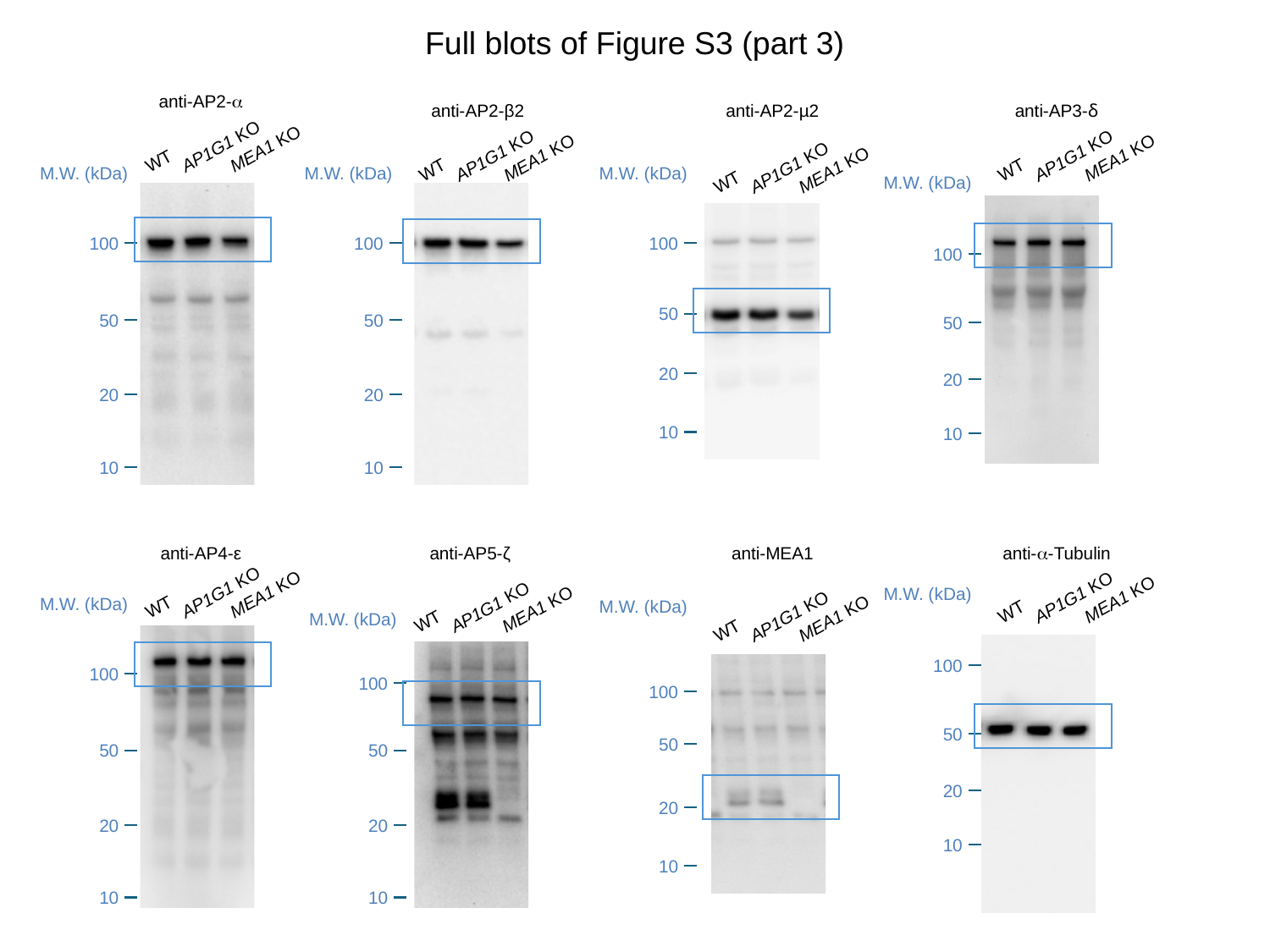

Full blots of Figure S3 (part 3)
anti-AP2-α
anti-AP2-β2
anti-AP2-µ2
anti-AP3-ẟ
AP1G1 KO
MEA1 KO
AP1G1 KO
MEA1 KO
AP1G1 KO
MEA1 KO
WT
AP1G1 KO
MEA1 KO
WT
WT
M.W. (kDa)
M.W. (kDa)
M.W. (kDa)
WT
M.W. (kDa)
100
100
100
100
50
50
50
50
20
20
20
20
10
10
10
10
anti-AP4-ε
anti-AP5-ζ
anti-MEA1
anti-α-Tubulin
AP1G1 KO
MEA1 KO
AP1G1 KO
MEA1 KO
M.W. (kDa)
AP1G1 KO
MEA1 KO
WT
WT
M.W. (kDa)
AP1G1 KO
MEA1 KO
M.W. (kDa)
WT
M.W. (kDa)
WT
100
100
100
100
50
50
50
50
20
20
20
20
10
10
10
10

## Slide 15
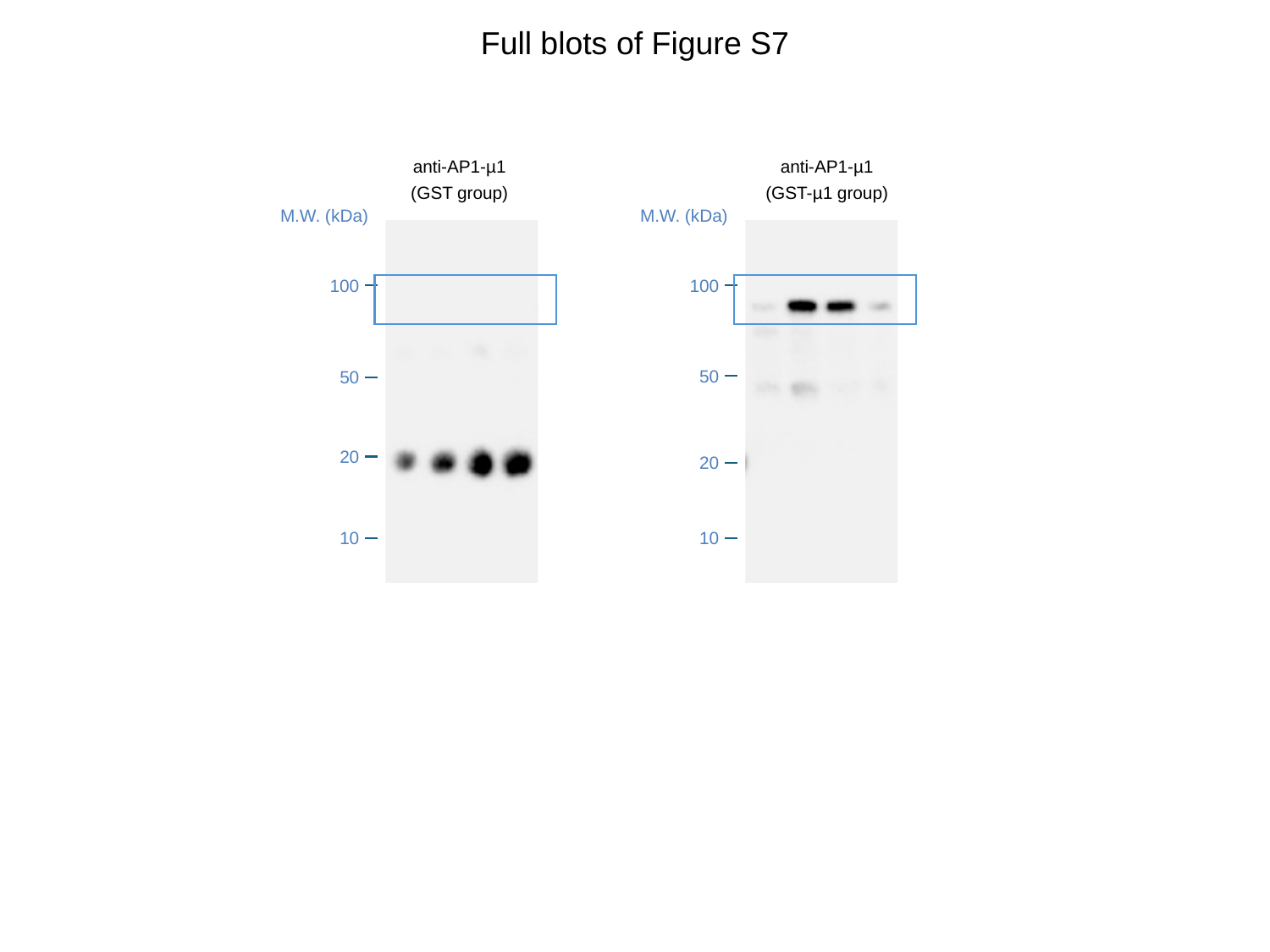

Full blots of Figure S7
anti-AP1-µ1
(GST group)
anti-AP1-µ1
(GST-µ1 group)
M.W. (kDa)
M.W. (kDa)
100
100
50
50
20
20
10
10

## Slide 16
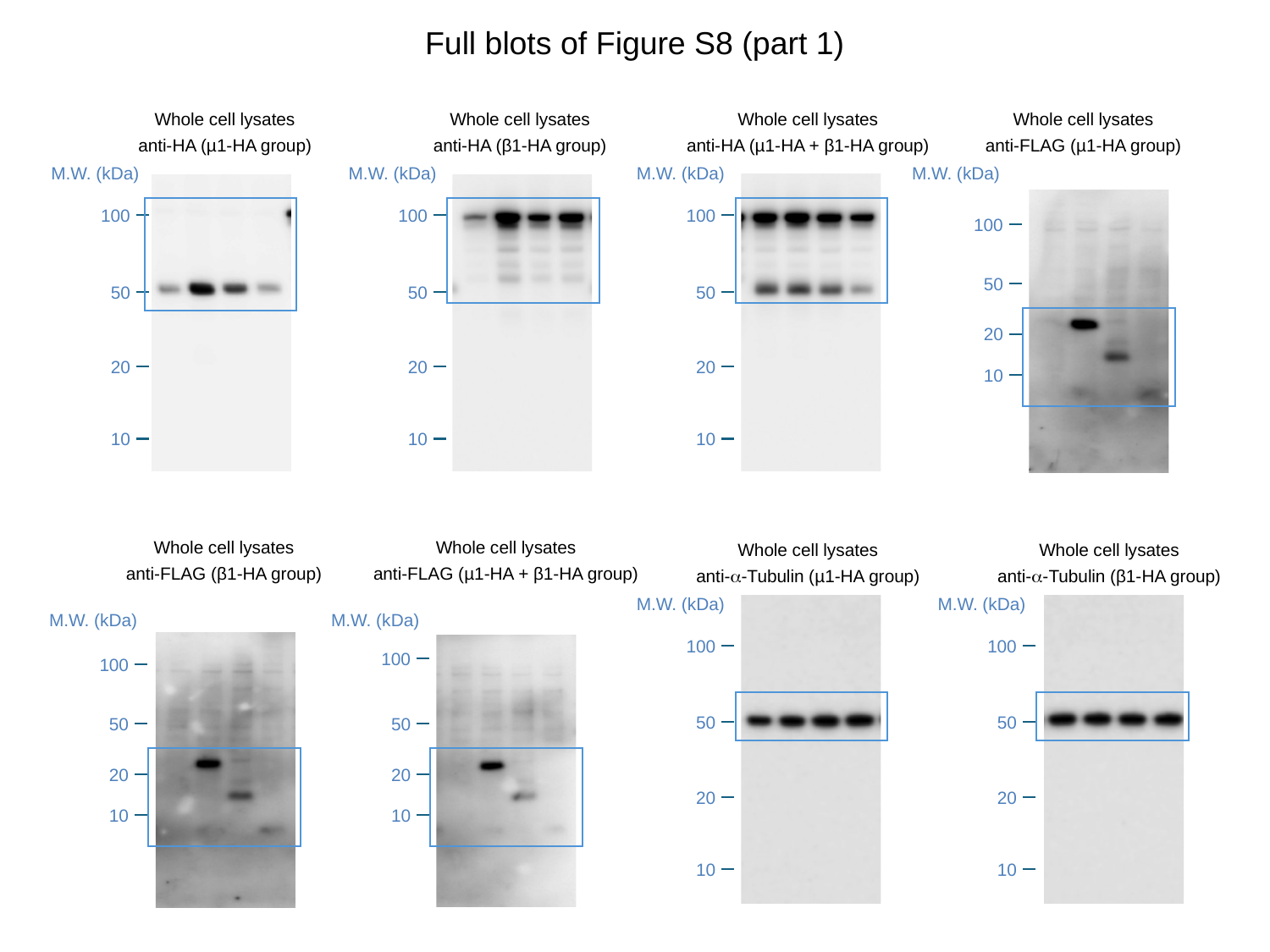

Full blots of Figure S8 (part 1)
Whole cell lysates
anti-HA (µ1-HA group)
Whole cell lysates
anti-HA (β1-HA group)
Whole cell lysates
anti-HA (µ1-HA + β1-HA group)
Whole cell lysates
anti-FLAG (µ1-HA group)
M.W. (kDa)
M.W. (kDa)
M.W. (kDa)
M.W. (kDa)
100
100
100
100
50
50
50
50
20
20
20
20
10
10
10
10
Whole cell lysates
anti-FLAG (β1-HA group)
Whole cell lysates
anti-FLAG (µ1-HA + β1-HA group)
Whole cell lysates
anti-α-Tubulin (µ1-HA group)
Whole cell lysates
anti-α-Tubulin (β1-HA group)
M.W. (kDa)
M.W. (kDa)
M.W. (kDa)
M.W. (kDa)
100
100
100
100
50
50
50
50
20
20
20
20
10
10
10
10

## Slide 17
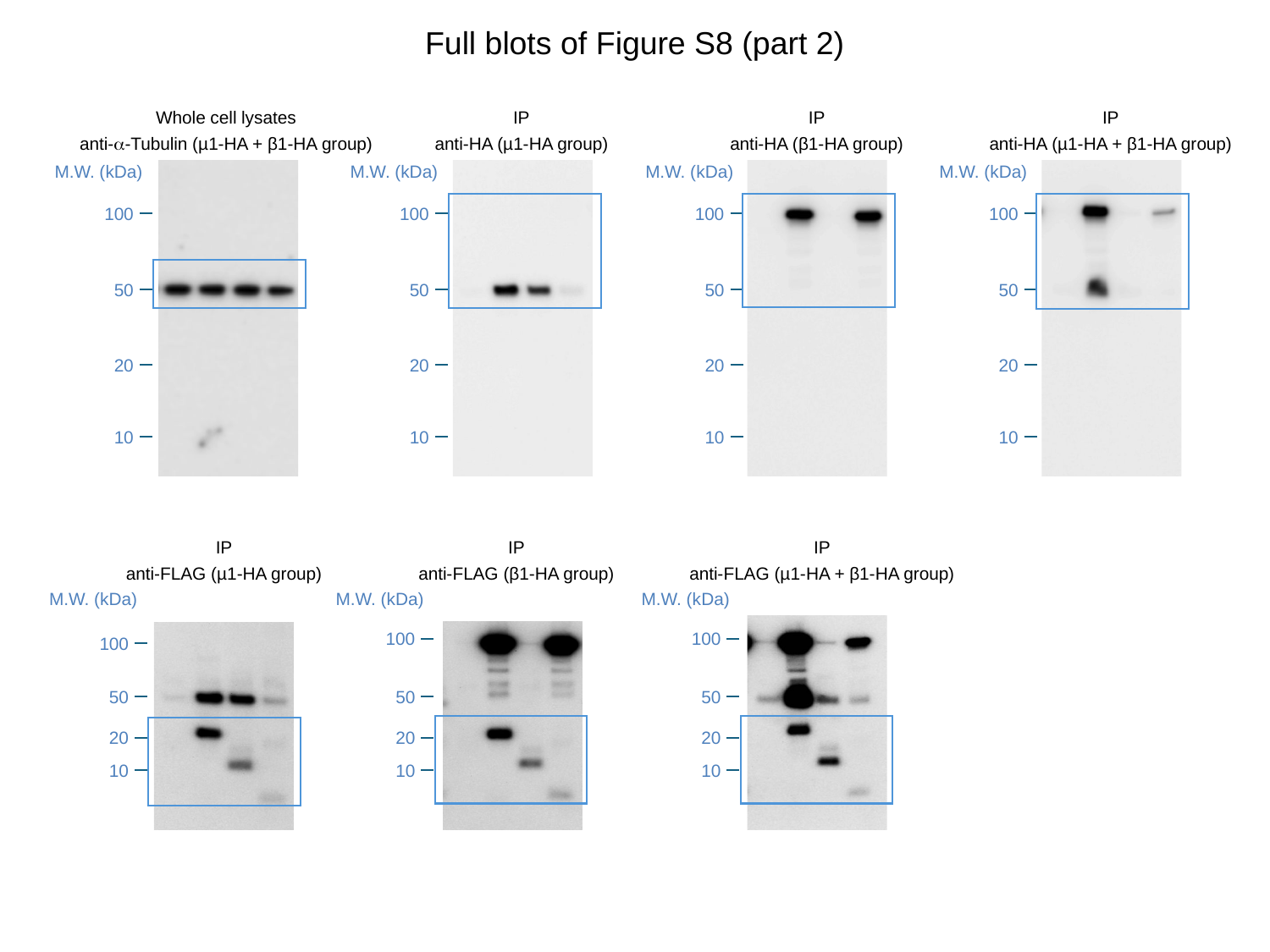

Full blots of Figure S8 (part 2)
Whole cell lysates
anti-α-Tubulin (µ1-HA + β1-HA group)
IP
anti-HA (µ1-HA group)
IP
anti-HA (β1-HA group)
IP
anti-HA (µ1-HA + β1-HA group)
M.W. (kDa)
M.W. (kDa)
M.W. (kDa)
M.W. (kDa)
100
100
100
100
50
50
50
50
20
20
20
20
10
10
10
10
IP
anti-FLAG (µ1-HA group)
IP
anti-FLAG (β1-HA group)
IP
anti-FLAG (µ1-HA + β1-HA group)
M.W. (kDa)
M.W. (kDa)
M.W. (kDa)
100
100
100
50
50
50
20
20
20
10
10
10

## Slide 18
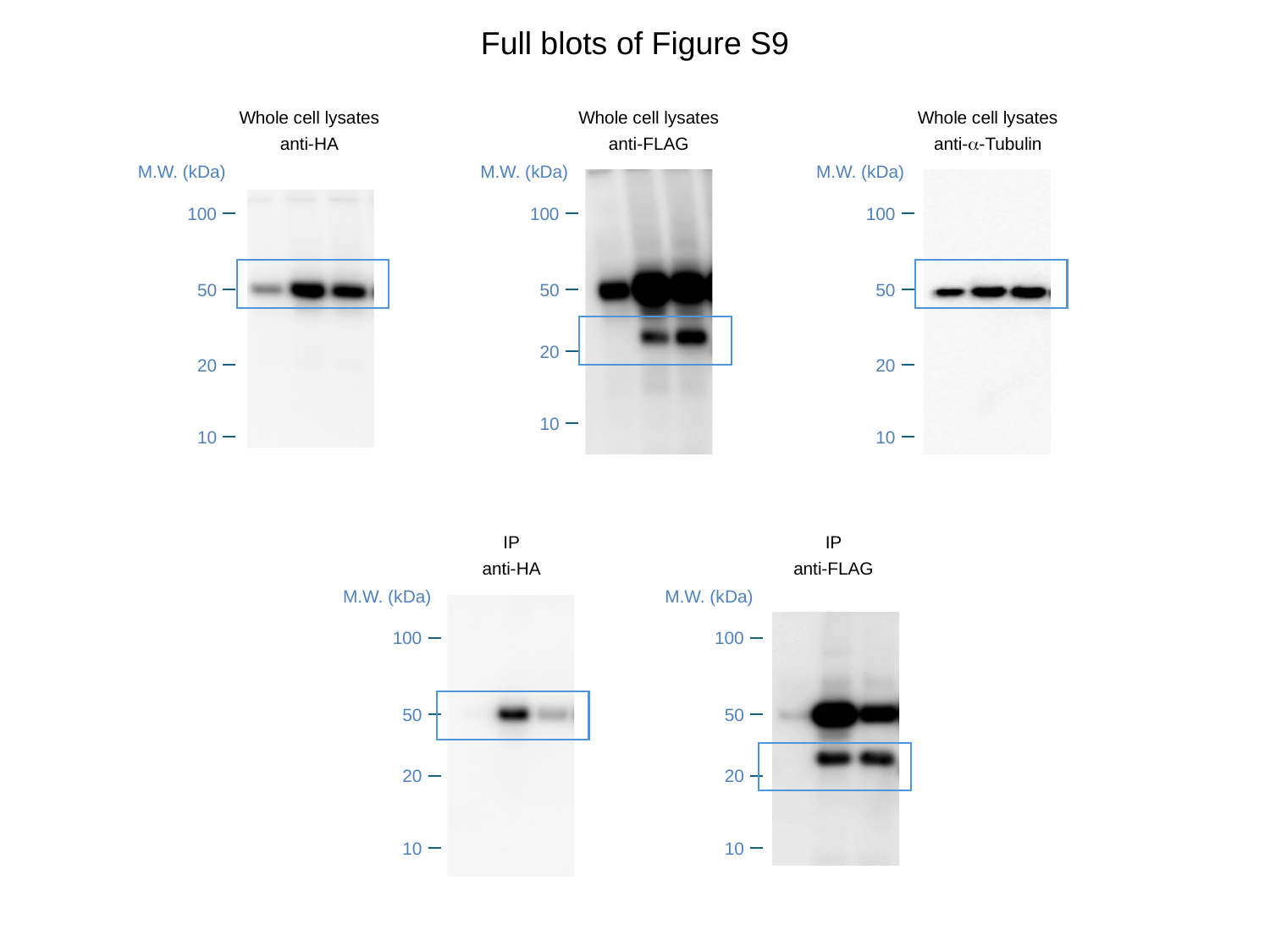

Full blots of Figure S9
Whole cell lysates
anti-HA
Whole cell lysates
anti-FLAG
Whole cell lysates
anti-α-Tubulin
M.W. (kDa)
M.W. (kDa)
M.W. (kDa)
100
100
100
50
50
50
20
20
20
10
10
10
IP
anti-HA
IP
anti-FLAG
M.W. (kDa)
M.W. (kDa)
100
100
50
50
20
20
10
10

## Slide 19
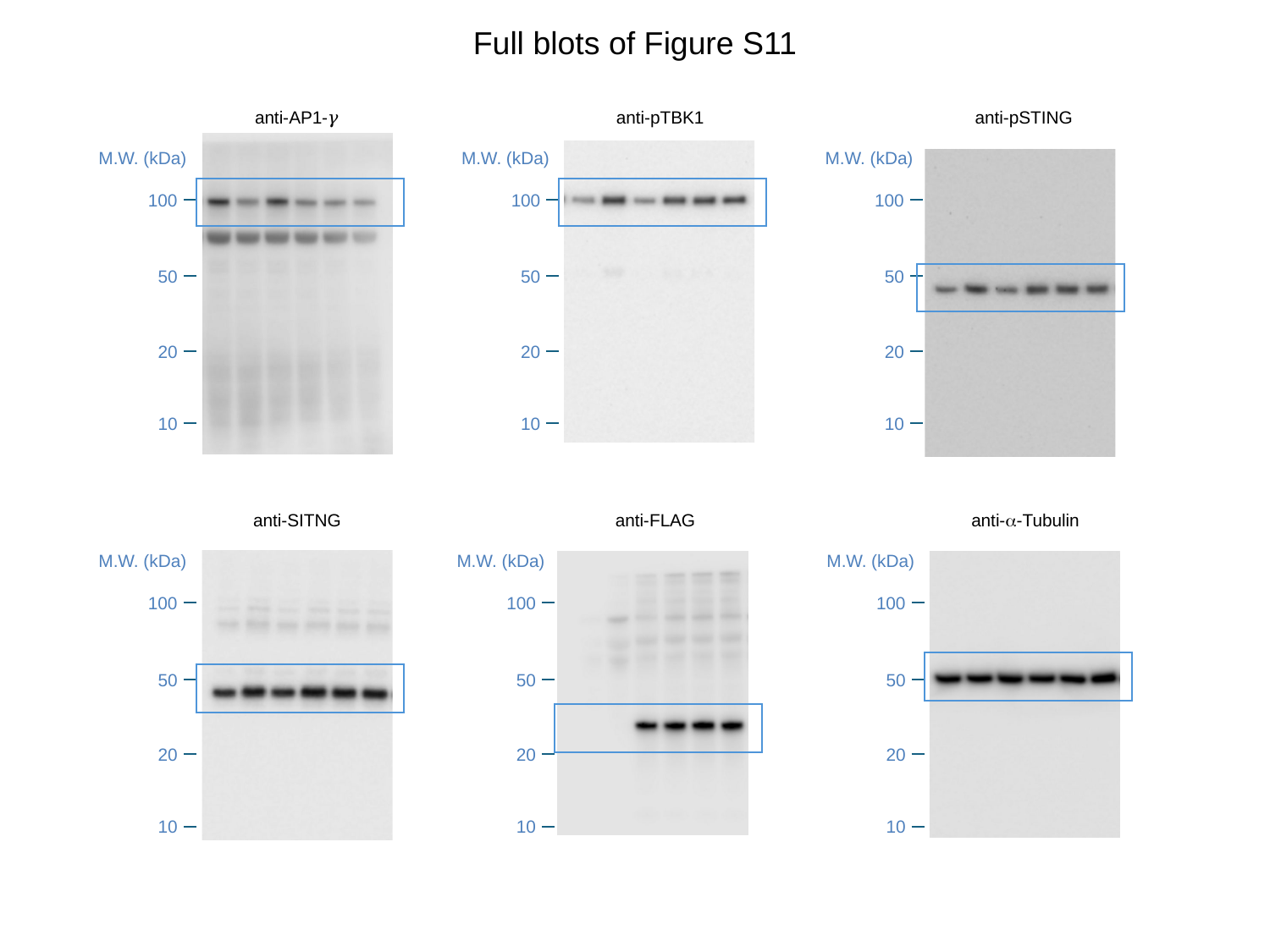

Full blots of Figure S11
anti-AP1-𝛾
anti-pTBK1
anti-pSTING
M.W. (kDa)
M.W. (kDa)
M.W. (kDa)
100
100
100
50
50
50
20
20
20
10
10
10
anti-SITNG
anti-FLAG
anti-α-Tubulin
M.W. (kDa)
M.W. (kDa)
M.W. (kDa)
100
100
100
50
50
50
20
20
20
10
10
10
